# Identification of specific prognostic markers for lung squamous cell carcinoma based on tumor progression, immune infiltration, and stem index

**DOI:** 10.3389/fimmu.2023.1236444

**Published:** 2023-09-29

**Authors:** Rihan Wu, Ru Ma, Xiaojun Duan, Jiandong Zhang, Kexin Li, Lei Yu, Mingyang Zhang, Pengxia Liu, Changshan Wang

**Affiliations:** ^1^ School of Life Science, Inner Mongolia University, Hohhot, China; ^2^ The Department of Oncology, Affiliated Hospital of Inner Mongolia Medical University, Hohhot, China; ^3^ School of Basic Medicine, Inner Mongolia Medical University, Hohhot, China

**Keywords:** LUSC, prognosis, biomarker, tumor microenvironment, cancer stem cell

## Abstract

**Introduction:**

Lung squamous cell carcinoma (LUSC) is a unique subform of nonsmall cell lung cancer (NSCLC). The lack of specific driver genes as therapeutic targets leads to worse prognoses in patients with LUSC, even with chemotherapy, radiotherapy, or immune checkpoint inhibitors. Furthermore, research on the LUSC-specific prognosis genes is lacking. This study aimed to develop a comprehensive LUSC-specific differentially expressed genes (DEGs) signature for prognosis correlated with tumor progression, immune infiltration,and stem index.

**Methods:**

RNA sequencing data for LUSC and lung adenocarcinoma (LUAD) were extracted from The Cancer Genome Atlas (TCGA) data portal, and DEGs analyses were conducted in TCGA-LUSC and TCGA-LUAD cohorts to identify specific DEGs associated with LUSC. Functional analysis and protein–protein interaction network were performed to annotate the roles of LUSC-specific DEGs and select the top 100 LUSC-specific DEGs. Univariate Cox regression and least absolute shrinkage and selection operator regression analyses were performed to select prognosis-related DEGs.

**Results:**

Overall, 1,604 LUSC-specific DEGs were obtained, and a validated seven-gene signature was constructed comprising FGG, C3, FGA, JUN, CST3, CPSF4, and HIST1H2BH. FGG, C3, FGA, JUN, and CST3 were correlated with poor LUSC prognosis, whereas CPSF4 and HIST1H2BH were potential positive prognosis markers in patients with LUSC. Receiver operating characteristic analysis further confirmed that the genetic profile could accurately estimate the overall survival of LUSC patients. Analysis of immune infiltration demonstrated that the high risk (HR) LUSC patients exhibited accelerated tumor infiltration, relative to low risk (LR) LUSC patients. Molecular expressions of immune checkpoint genes differed significantly between the HR and LR cohorts. A ceRNA network containing 19 lncRNAs, 50 miRNAs, and 7 prognostic DEGs was constructed to demonstrate the prognostic value of novel biomarkers of LUSC-specific DEGs based on tumor progression, stemindex, and immune infiltration. In vitro experimental models confirmed that LUSC-specific DEG FGG expression was significantly higher in tumor cells and correlated with immune tumor progression, immune infiltration, and stem index. *In vitro* experimental models confirmed that LUSC-specific DEG FGG expression was significantly higher in tumor cells and correlated with immune tumor progression, immune infiltration, and stem index.

**Conclusion:**

Our study demonstrated the potential clinical implication of the 7- DEGs signature for prognosis prediction of LUSC patients based on tumor progression, immune infiltration, and stem index. And the FGG could be an independent prognostic biomarker of LUSC promoting cell proliferation, migration, invasion, THP-1 cell infiltration, and stem cell maintenance.

## Graphical Abstract

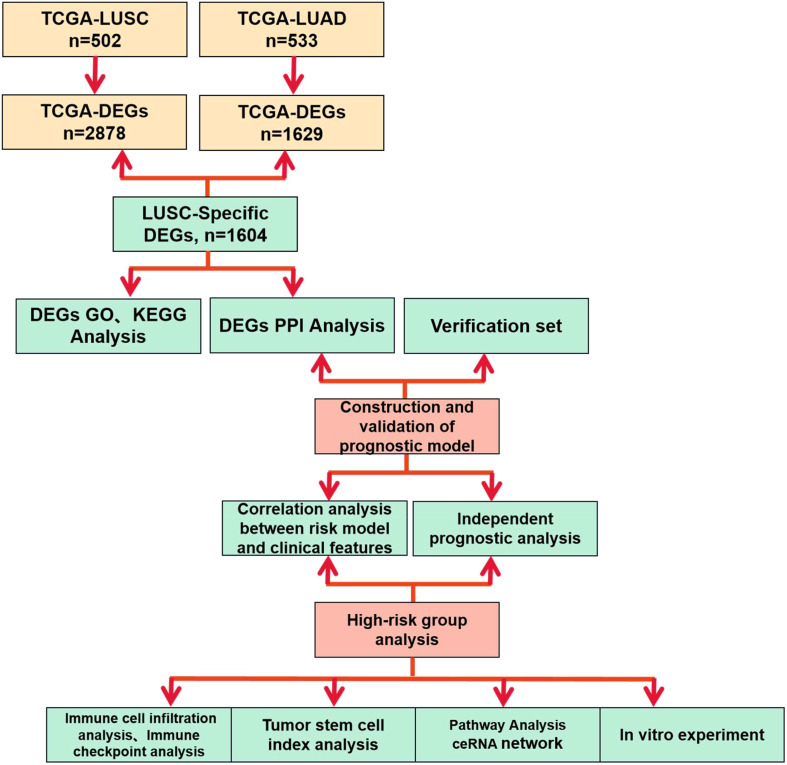

## 1 Introduction

Lung cancer is heterogeneous and fatal, with non-small cell lung cancer (NSCLC) as its main pathological subtype. Lung squamous cell carcinoma (LUSC) and lung adenocarcinoma (LUAD) are the primary subtypes of NSCLC (1); however, they differ in many aspects, including the origin of cells, genetic variation, epigenetics, and the outcome of antineoplaston drugs (2). Despite tremendous advances in diagnosis and treatment, including molecular targeted therapeutics and immunotherapy, the clinical outcomes of LUSC remain unsatisfactory (3). Patients with LUSC are often diagnosed in an advanced stage when existing therapy cannot be administered in a timely manner (4). LUSC patients are also not as sensitive as LUAD patients to chemotherapy, radiotherapy, and tumor immunotherapy. In addition, the prognosis of LUSC is poor, with an estimated 5-year survival rate of <15% (5). Therefore, distinguishing LUSC from LUAD is important to identify effective prognostic biomarkers.

Although studies based on the whole genome (6, 7), epigenetics (8), cancer stem cells (CSCs) (9, 10), and tumor microenvironment (TME) (11) have analyzed differentially expressed genes (DEGs) in LUSC and LUAD, research on the LUSC-specific prognosis genes is lacking. A previous study (4), involving 178 LUSC cases, conducted using the Cancer Genome Atlas (TCGA) Research Network reported complex genomic alterations in LUSC, including significant copy number alterations, which peaked for SOX2, PDGFRA/KIT, EGFR, FGFR1, CCND1, and CDKN2A. In LUSC, CDKN2A/RB1, NFE2L2/KEAP1, squamous differentiation genes, and PI3K/Akt were significantly altered. TP53 is the most commonly mutated gene with a mutation frequency of > 80% in LUSC. The overexpression and amplification of genes, SOX2 and TP63, are spectrum factors of LUSC (12). Despite progress in research on biomarkers for LUSC, oncology targets are rare. Recent studies on genetic biomarkers for LUSC have focused on a single gene based on the cognitions of CSCs and the TME in cancer progression, as well as drug resistance and response to immune checkpoint blockade. Traditional methods use differential expression detection to identify potential biomarkers but may miss out on useful genes. As the occurrence and development of malignant tumors is a long-term complex process involving genomic changes, the interaction between tumor cells and their immune microenvironment, and the participation of tumor stem cells, the behavior cognition of malignant tumors warrants extensive research.

Therefore, in this study, we aimed to compare DEGs of LUSC with LUAD using biological information analytical methods based on prognostic risk factors, including tumor invasion, metastasis, survival, immune infiltration, and tumor stem cell-related genes. DEGs in LUSC were employed to generate a risk model to evaluate the prognostic value of characteristic genes for possible prognostic indicators or therapeutic targets for LUSC. We further explored the associations between the specific prognostic markers FGG and tumor progression, immune invasion, and the tumor cell stem index to identify potential LUSC-specific survival prognostic biomarkers and therapeutic targets.

## 2 Methods

### 2.1 Data processing

We first retrieved LUSC (n=502) and LUAD (n=533) RNA sequencing datasets, and the clinical information of corresponding LUSC patients and 59 healthy volunteers from the TCGA database (https://portal.gdc.cancer.gov/).

### 2.2 Differentially expressed genes

The “limma” package was selected for DEGs analysis in TCGA-LUSC and TCGA-LUAD cohorts. For processing, a |log2 (fold change)| > 0.5 and adjusted P-value < 0.05 were considered the cut-off criteria for screening the DEGs between the tumor and normal samples. The “heatmap” package of the R program was used to generate a heatmap of the top 100 DEGs. Additionally, we employed a Venn diagram to indicate the specific DEGs in LUSC.

### 2.3 Functional enrichment analyses

The Gene Ontology (GO) and Kyoto Encyclopedia of Genes and Genomes (KEGG) pathway enrichment analyses of LUSC-specific DEGs were conducted with the “clusterProfiler” package of the R software; P < 0.05 was the statistical significance threshold. The bubble plot, circle graph, and heat map were plotted using R to visualize the top enrichment GO terms and KEGG networks. To explore the pathways and GO functions of unique differential genes in LUSC, the R “clusterProfiler” package was used for enrichment analysis based on KEGG and GO to search for common functions among DEGs, as well as related pathways of several genes. Statistical methods were used to calculate the cumulative hypergeometric distribution to analyze, within a group of genes, whether overpresentation occurs on a functional node, as follows:


$$P(\text{X}>\text{q})=1-\sum_{x=1}^{q}\frac{{(}_{x}^{n}){(}_{M-x}^{N-n})}{{(}_{M}^{N})}$$

where ‘N’ is the total gene number within the annotation system, ‘n’ is the gene number annotated by the node or pathway itself to be examined, ‘M’ is the size of the DEGs set, and ‘x’ is the number of intersections between gene sets and nodes or pathways.

### 2.4 Protein–protein interaction axis for LUSC DEGs

The association between LUSC-specific DEGs was predicted using STRING (https://string-db.org). The PPI axis was visualized with the Cytoscape software at a confidence of 0.9. In the PPI network, the individual DEG’s adjacent node numbers were computed, and the top 20 DEGs were displayed using a bar plot according to the number of adjacent nodes. Weighted gene co-expression network analysis (WGCNA) was conducted to screen out relevant modules and hub genes, which were used to develop the prognostic signature. TCGA and GTEx data based gene expression profiling interactive analysis (GEPIA) was used to predict gene interactive and customizable functions.

### 2.5 Construction and validation of a gene signature constructed from LUSC-specific DEGs

Based on the number of connections, the top 100 LUSC-specific DEGs were selected for subsequent analyses. We extracted the expression data of the 100 LUSC-specific genes from TCGA-LUSC patients and combined them with the clinical information of corresponding patients. The corresponding patients with TCGA-LUSC were randomly divided into a training cohort (TC, n = 336) and a validation cohort (VC, n = 145) in a 7:3 ratio. To identify prognostic genes in LUSC, we conducted a univariate Cox regression analysis on 100 LUSC-specific DEGs. Those with a P < 0.05 were considered correlated with the LUSC prognosis. Subsequently, we used the least absolute shrinkage and selection operator (LASSO) and Cox regression analyses to obtain the genetic profile with the most significant prognosis from the LUSC-specific DEGs within the TCGA-LUSC patient population via the “glmnet” package in R. Individual patient risk score (RS) was computed based on the levels of the prognostic signature genes and the associated coefficients obtained from the LASSO-Cox regression model. LUSC patients were categorized into high risk (HR) and low risk (LR) cohorts based on the median RS. The overall survival (OS) of the different risk cohorts was analyzed using Kaplan–Meier analysis with the log-rank test using the “Kaplan–Meier survival” package in R. Moreover, the time dependent receiver operating characteristic (ROC) curve was generated via the “survival ROC” package in R to demonstrate the effectiveness of the genetic profile. To analyze the relationship between predictive and response variables, we employed the uni- and multivariate Cox regression analyses.

### 2.6 Single sample gene set enrichment analysis

The relative tumor infiltration levels of 29 immune-linked gene sets (16 immune cell types and 13 immune-linked pathways) between HR and LR groups were quantified by ssGSEA. The analysis was conducted using the “gsva” R package. Comparisons between the HR and LR cohorts were carried out via the Wilcoxon test.

### 2.7 Tumor stem cell index analysis

The mRNA expression based stemness index (mRNAsi) and epigenetically regulated mRNAsi (EREG-mRNAsi) in LUSC samples were computed using the OCLR algorithm for research on gastric cancer (13) and NSCLC (14). Subsequently, the differences in mRNAsi and EREG-mRNAsi between the HR and LR cohorts were compared using the Wilcoxon test. The two independent stemness indices range from 0 to 1, with a value closer to 1 suggesting stronger characteristics of CSCs.

### 2.8 Generation of the ceRNA axis

Differentially expressed lncRNAs (DE-lncRNAs) between tumor and healthy samples were recognized as follows: |log2 (fold change)| > 1 and P-value < 0.05. The target miRNAs of lncRNAs were estimated via the miRcode database (http://www.mircode.org/), and the target miRNAs of prognostic DEGs were estimated via the miRanda database (http://www.microrna.org/microrna/home.do). The common miRNAs predicted by the miRcode and miRanda databases, as well as the corresponding lncRNA and prognostic DEGs, were input into Cytoscape software to construct a ceRNA network.

### 2.9 Cell culture

LTEP-s, BEAS-2B, and NCI-H520 cells were purchased from the American Type Culture Collection (ATCC) and cultured in DMEM (HyClone, USA). NCI-H520 cells were cultured in RPMI-1640 medium (Biological Industries, USA). All cells were supplemented with 10% fetal bovine serum (FBS; Biological Industries, USA) and 1% penicillin/streptomycin (Sigma, USA) and cultured under standard culture conditions (37 °C, 5% CO_2_) in culture medium recommended by the ATCC.

### 2.10 RNA extraction and real-time polymerase chain reaction assay

Total RNA was extracted using TRIzol Reagent (Invitrogen, Carlsbad, CA) according to the manufacturer’s instructions. cDNA was synthesized using random primers and the PrimeScript RT Reagent Kit (Takara, China). Real-time polymerase chain reaction (qPCR) was performed using SYBR Premix Ex Tag (Takara, China). The PCR conditions were as follows: 95 °C for 15 s followed by 40 cycles of 95 °C for 5 s and 60 °C for 30 s. β-actin was used as the internal control. The primer sequences for real-time PCR are listed in **Table 1**.

**Table 1:** Primer sequences for real-time PCR used in the study.

Primer name	Primer sequences (5`–3`)	
FGG	Forward Primer	TTATTGTCCAACTACCTGTGGC
	Reverse Primer	GACTTCAAAGTAGCAGCGTCTAT
FGA	Forward Primer	AGACATCAATCTGCCTGCAAA
	Reverse Primer	AGTGGTCAACGAATGAGAATCC
JUN	Forward Primer	TCCAAGTGCCGAAAAAGGAAG
	Reverse Primer	CGAGTTCTGAGCTTTCAAGGT
CPSF4	Forward Primer	CATCGGGGTCATGCAGAGTC
	Reverse Primer	CTCGCCACACTTGTAACAGGT
HIST1H2BH-1F	Forward Primer	TCACCTCCAGGGAGATCCAG
	Reverse Primer	TTTGGGTTTGAACATGCGTCC
C3	Forward Primer	GGGGAGTCCCATGTACTCTATC
	Reverse Primer	GGAAGTCGTGGACAGTAACAG
CST3	Forward Primer	GTCGGCGAGTACAACAAAGC
	Reverse Primer	CACCCCAGCTACGATCTGC

### 2.11 Cell transfection

Small-interfering RNA (siRNA) oligonucleotides for FGG were designed and synthesized by Jima Bio (Suzhou, China). The primer sequences for the siRNAs are listed in **Table 2**. Transient transfection was performed using Lipofectamine 2000 Reagent (Invitrogen, USA) according to the manufacturer’s instructions. After transfection for 48 h, cells were used for functional assays, including migration, invasion, RNA extraction, and Western blotting.

**Table 2:** Primer sequences for siRNA used in the study.

Primer name	Primer sequences (5`–3`)	
FGG-homo-935	sense	CCUACUGGCACAACAGAAUTT
	antisense	AUUCUGUUGUGCCAGUAGGTT
FGG-homo-768	sense	GCGGGCUUUACUUUAUUAATT
	antisense	UUAAUAAAGUAAAGCCCGCTT
FGG-homo-1361	sense	GGUUAUGAUAAUGGCAUUATT
	antisense	UAAUGCCAUUAUCAUAACCTT

### 2.12 Cell proliferation assay

Cells were seeded in 96-well plates at 1 × 10^3^ cells per well and cultured in a final volume of 100 μL of culture medium supplemented with 10% FBS. The cell proliferation was determined using CCK-8. After incubation for 24, 48, 72, and 96 h, 20 uL of CCK-8 reagent was added for 3 h, and the absorption at a wavelength of 490 nm was determined.

### 2.13 Cell cycle assay

The cell suspension was diluted to 5×10^6^ cells/mL, the supernatant was removed, and 70% 500 μL of cold ethanol was added and placed in a refrigerator at 4°C for 2 h. The cell pellet was mixed with 100 μL RNaseA (Solarbio, China) and placed in a 37°C water bath for 30 min; PI staining buffer was added in the dark for 30 min at 4°C. Red fluorescence at 488 nm was detected using flow cytometer.

### 2.14 Cell apoptosis assay

The cell culture medium was collected into a centrifuge tube. The cells were digested with Edta-free pancreatic enzymes and added into the cell culture medium, centrifuged, and precipitated. The cells were then re-suspended with 1 mL PBS precooled at 4 °C and the precipitated cells were centrifuged again. The cells were re-suspended with 1x binding buffer and the concentration was adjusted to 5 × 10^6^/mL; 100 μL cell suspension was added to a 5 mL flow tube, mixed with 5 μL Annexin V/FITC (Solarbio, China), and incubated at room temperature for 5 min in the dark. A total of 5 μL propyl iodide solution (PI) and 400 μL PBS were added for immediate flow detection.

### 2.15 Wound healing assay

Cells were placed in 12-well plates. When cells grew to 90–95% confluence, cell monolayers were wounded by scratching with plastic micropipette tips and washed twice with PBS. The cells were rinsed with PBS and cultured in DMEM or RPMI 1640 supplemented with 1% FBS. Images of the different stages of wound healing were obtained via microscopy at 0, 24, and 48 h. Relative cell motility was quantified using Image-Pro Plus.

### 2.16 Transwell migration and invasion assay

Cell migration and invasion assays were performed in 24-well plates with 8-μm-pore size chamber inserts (Corning, USA). For the migration assays, 5 × 10^4^ cells in 200 μL of serum-free culture medium were seeded into each well of the upper chamber with the noncoated membrane, and 800 μL of medium supplemented with 10% FBS was added to the lower chamber. For invasion assays, 1 × 10^5^ cells in 200 μL of serum-free culture medium were seeded into each well of the upper chamber with the Matrigel-coated membrane, while 800 μL of medium supplemented with 10% FBS was added to the lower chamber. Cells that migrated through the membrane were fixed with 100% methanol, stained with 0.1% crystal violet for 30 min, imaged, and counted under a light microscope (Olympus, Japan).

### 2.17 Western blot assay

Cells grown in 6-well plates were lysed on ice using RIPA buffer. The lysis mixtures were centrifuged, and the supernatants were collected. Total protein was separated using SDS-polyacrylamide gel electrophoresis and transferred onto PVDF membranes (Millipore, USA). After blocking the membranes with non-fat milk, the membranes were incubated overnight with the following primary antibodies: anti-N-cadherin (1:1,000), anti-E-cadherin (1:1,000), anti-GAPDH (1:1,000) (Abcam, UK). The membranes were then incubated with horseradish peroxidase-conjugated secondary antibodies (1:2,000). The analysis was performed using an enhanced chemiluminescence system (Bio-Rad, USA). Binding was analyzed using Image J 6.0.

### 2.18 THP-1 cell infiltration

THP-1 cells were seeded at 1×10^6^ per well in 6-well plates and treated with PMA (100 nmol; Sigma-Aldrich, USA) for 48 h. M1 macrophages were polarized by incubation with INF-γ (20 ng/mL; R&D System, USA) and LPS (100 ng/mL; Sigma, USA) for 48 h.

After transfection with si-NC or si-FGG in the absence or presence of coculture, a cell migration assay was conducted using 24-well Transwell plates (8.0 μm; Corning, NY, USA). The macrophages or cancer cells (5×10^4^, LTEPs-si-NC, LTEPs-si-FGG) were planted into the upper chambers, while 600 µL RPMI 1640 containing 10% FBS were placed into the lower chambers. Thereafter, the Transwell plates were incubated at 37 °C, 5% CO_2_ for 48 h, fixed in 4% formaldehyde for 30 min, and stained with 0.01% crystal violet. Non-migrating cells were carefully removed with a cotton swab, while cells that migrated to the lower chambers were counted under a microscope.

### 2.19 Statistical analysis

All data analyses were conducted using the R language (version 3.5.1). The levels of immune checkpoint genes between the HR and LR cohorts were compared using the Wilcoxon test. Uni- and multivariate analyses were employed to screen for stand-alone prognostic markers for LUSC survival. P < 0.05 was set as the significance threshold.

## 3 Results

### 3.1 Identification of specific DEGs for LUSC

**Figure 1:**
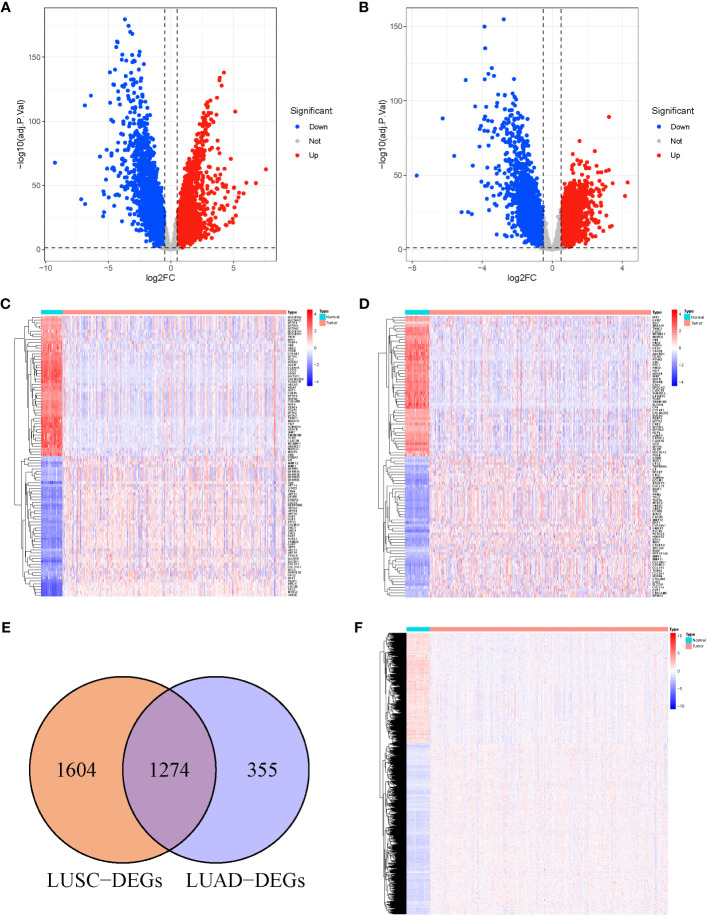
**(A)** Volcano Plots of 2,878 LUSC-DEGs. **(B)** Volcano Plots of 1,629 LUAD-DEGs. Multiples of the abscissa difference (Tumor/Normal) taken the logarithm of 2 and the ordinate representation of -log_10_(adj.P.Val). Each dot represents a gene. Red dots indicate gene upregulation (Tumor vs. Normal samples), blue dots indicate downregulation (Tumor vs. Normal samples), and gray dots indicate no significant differences in expression. **(C)** The heat map of the top 100 DEGs in LUSC. **(D)** The top 100 DEGs LUAD. The abscissa direction represents the DEGs, while the vertical direction represents the samples. Colors indicate normalized differential expression; high and low expressions are shown in red and blue, respectively. **(E)** The Venn diagram of 1,604 LUSC-specific DEGs calculated by subtraction of LUSC-DEGs and the cross-section of LUSC and LUAD DEGs. **(F)** The heat map of LUSC-specific DEGs. The abscissa direction indicates the DEGs, while the vertical direction indicates the samples. Colors indicate normalized differential expression; red represents elevated levels, and blue represents reduced levels.

We analyzed DEGs between tumor and normal samples in TCGA-LUSC and TCGA-LUAD cohorts. Overall, 2,878 DEGs (1,466 upregulated and 1,412 downregulated) were identified in LUSC, relative to normal samples (**Figure 1A**). In addition, 1,629 DEGs were identified in LUAD, among which, 714 were highly expressed and 915 were scarcely expressed (**Figure 1B**). The top 100 DEGs in LUSC (**Figure 1C**) and LUAD (**Figure 1D**) are shown in the heat maps. We further applied an online Venn diagram to identify LUSC-specific DEGs (**Figure 1E**). Consequently, 1,604 specific DEGs for LUSC were obtained, as shown in a heat map (**Figure 1F**).

### 3.2 FEA and PPI analysis of the novel biomarkers in LUSC

**Figure 2:**
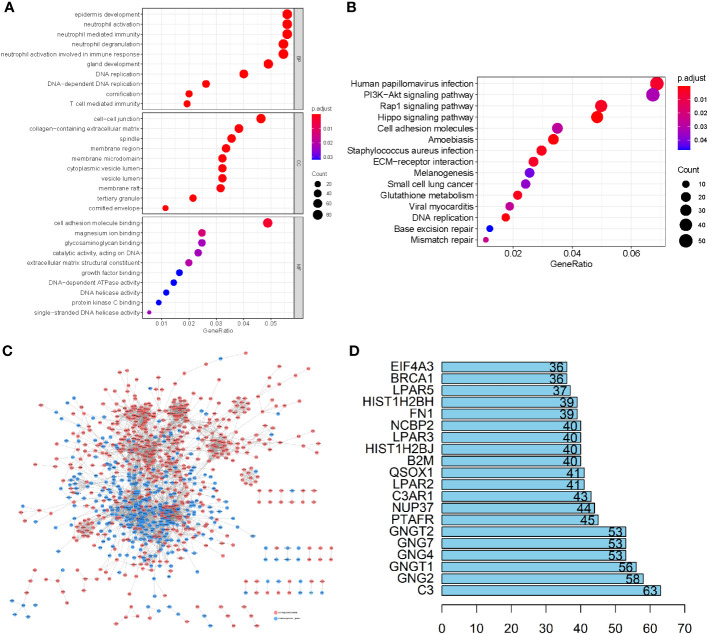
**(A)** GO enrichment analysis of the LUSC-specific DEGs. **(B)** The KEGG analysis of LUSC-specific DEGs. The ordinate and abscissa are the GO pathway sorted by the P-value and gene proportion. The shades of color denote the P-value, while the dot sizes represent the number of participating genes. **(C)** A PPI network containing 1,604 nodes and 14,209 edges further revealed the interactions of these LUSC-specific DEGs, where lines represent the interactions between them, red nodes refer to elevated gene expression, and blue nodes refer to diminished gene expression. **(D)** The bar plot of the top 20 DEGs in LUSC-specific DEGs.

To elucidate the physiological activities of these LUSC-specific DEGs, GO and KEGG enrichment analyses were carried out. GO terms revealed that these LUSC-specific DEGs were markedly enriched in immune-linked biological systems such as T cell-mediated immunity, immune response-related neutrophil activation, neutrophil degranulation, neutrophil-based immunity, and neutrophil activation (**Figure 2A**). KEGG analysis revealed that the LUSC-specific DEGs were associated with melanogenesis, small-cell lung cancer, the PI3K-Akt axis, viral myocarditis, human papillomavirus infection, ECM-receptor association, the Rap1 signaling pathway, Staphylococcus aureus infection, and glutathione metabolism (**Figure 2B**). PPI interaction networks containing 1,604 nodes and 14,209 edges further revealed the interactions between these LUSC-specific DEGs (**Figure 2C**). The top 20 DEGs are displayed in a bar plot based on the quantity of adjacent nodes (**Figure 2D**). The top 100 genes of connectedness were obtained using a collateral analysis. The genes with the top 100 connectedness were single factors. Then, Cox and LASSO regression analyses were employed for risk model construction. WGCNA was used to analyze the hub genes’ biological behavior, and the correlation between alteration in hub gene expression and clinical characteristics was confirmed via external data from the GEPIA Database (http://gepia.cancer-pku.cn/). The results of WGCNA and GEPIA for hub genes suggested that all hub genes were significantly elevated in tumor tissues. Following the adjustment of confounding factors, we developed a prognostic profile using three genes with remarkable predictive ability.

### 3.3 Prognostic signature of LUSC-specific DEGs

**Figure 3:**
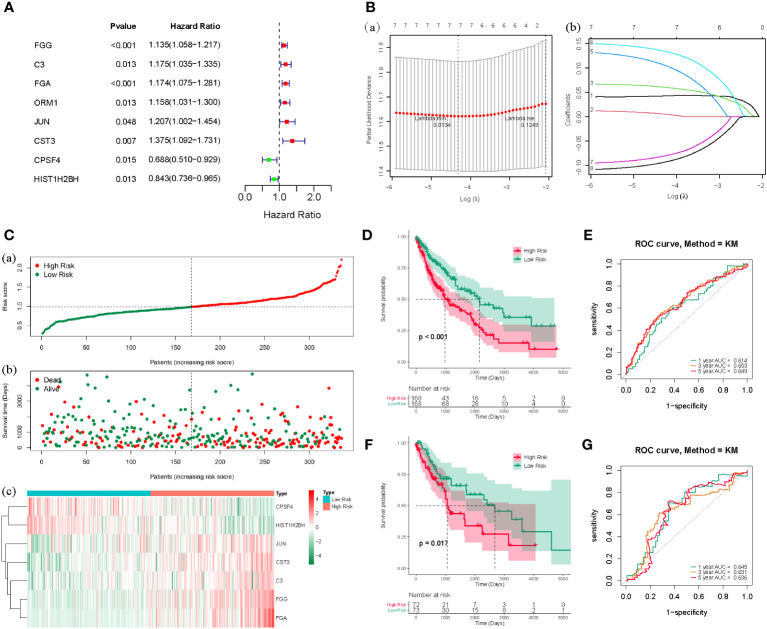
**(A)** The forest map of the eight risk genes (FGG, C3, FGA, CRM1, JUN, CST3, CPSF4, and HIST1H2BH) using univariate analysis. **(B)**, (a) LASSO analysis, where the screened characteristic gene ordinate is the gene coefficient; (b) the abscissa is the log(Lambda), and the ordinate denotes cross-validation error. In the analysis, we identified the position with the minimum error of cross-validation. In **(B)**, the dotted line on the left represents the position with the minimum error of cross-validation. Based on the position (lambda.min), we determined the associated horizontal coordinate log(Lambda) and the number of characteristic genes (shown above); we also found the optimal log(Lambda) value and the associated gene and its coefficient in the left figure **(A)**. **(C)** The risk curve and the distributions of OS status of the seven-gene TC (P < 0.05). The risk score (RS) of the TC in high- (HR) and low-risk (LR) cohorts (a), the OS status (b), and the heat map (c) are shown. The figure above (a) is consistent with the abscissa of the middle figure (b), indicating that RSs rose from left to right. The ordinate represents the RS and survival time, while the dotted line represents the median RS and the corresponding number of patients. Below (c) is the gene expression heat maps in the HR and LR cohorts. **(D)** The OS curve based on the HR and LR cohorts. **(E)** ROC curve of the seven-gene set in TCGA-LUSC training cohort (TC) 1-3-5-years OS. **(F)** TCGA-LUSC validation of survival curves for concentrated HR and LR cohorts. **(G)** ROC curves for 1-3-5-years OS in TCGA-LUSC validation cohort (VC).

Based on the counts of connections, the top 100 LUSC-specific DEGs in the PPI network were selected for further analysis. To identify the prognostic genes in LUSC, we further employed a univariate analysis of the 100 LUSC-specific DEGs. Eight were associated with the prognosis of patients with LUSC (P < 0.05); univariate Cox regression analysis results are shown in **Supplementary Table 1**. FGG, C3, FGA, ORM1, JUN, and CST3 served as risk hazards (HR > 1), whereas CPSF4 and HIST1H2BH served as a protective function (HR < 1) in LUSC (**Figure 3A**). LASSO analysis was employed to improve the robustness of the eight LUSC-specific DEGs. Eight genes carrying a P-value < 0.05 in the univariate Cox analysis were used to construct a LASSO regression. To reduce the feature dimension, we used the R software’s “glmnet” package, set the parameter family as Cox, realized LASSO logistic regression, selected strong correlation features, and obtained the two graphs depicted in **Figure 3**; one is the graph of gene coefficient, and the other is the error graph of cross-validation. As shown in **Figure 3B**, the seven characteristic genes with a lambda.min of 0.0134 were FGG, C3, FGA, JUN, CST3, CPSF4, and HIST1H2BH. The seven genes and their corresponding coefficients were selected as the most prognostic gene signatures in LUSC. We further calculated the RS for individual patients with LUSC using the expression of the seven prognostic genes and associated coefficients retrieved from the LASSO-Cox analysis; LASSO analysis was then employed for characteristic genes and coefficients screening, as shown in **Supplementary Table 2**. Subsequently, the median of the RSs was utilized as a standard to separate the LUSC patients into HR and LR cohorts in both the TC and VC. The risk curve and distribution of OS status are shown in **Figure 3C**. Moreover, the expression patterns of the seven prognostic genes in the HR and LR cohorts verified the prognostic value of the seven markers. **Figure 3C** consists of three parts: upper (a), middle (b), and lower (b), all of which demonstrate that the HR cohort exhibited an elevated survival RS. Notably, the Kaplan–Meier analysis indicated that LR LUSC patients exhibited a markedly higher survival probability, compared to the HR cohort (**Figure 3D**; P < 0.05). The results of ROC analysis further tested the TC, which showed that this genetic profile could effectively estimate the OS of LUSC (**Figure 3E**).

The VC was also tested, and the risk curve and distribution of OS status are shown in **Supplementary Figure 1**. The survival and ROC curves of VC are shown in **Figures 3F, G**.

### 3.4 The seven-gene signature of LUSC represents an independent stand-alone prognostic value

**Figure 4:**
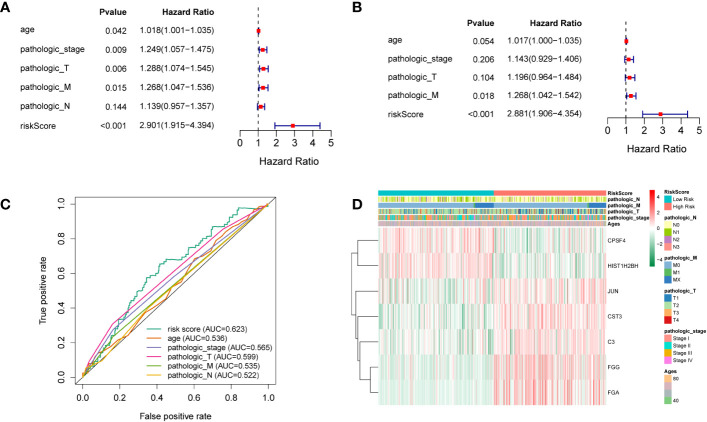
**(A)** Univariate analysis. **(B)** Multivariate analysis. **(C)** ROC curves of multiple indicators. **(D)** Heat maps of different clinicopathological features of TCGA-LUSC.

To elucidate whether the prognostic gene profile was independent of clinicopathological features such as age, pathological stage, and TNM stage, univariate and multivariate analyses were conducted. Univariate analysis revealed that age, as well as pathologic, pathologic T, and pathologic M stages, were strongly correlated with LUSC patients’ OS (**Figure 4A**). Multivariate analysis based on the above clinicopathological characteristics further revealed that the RS was directly correlated with OS (**Figure 4B**; P < 0.001). The predictive efficiency of these clinicopathological characteristics was evaluated using ROC analysis, and the RS was employed as a predictor stand-alone indicator of LUSC outcome (**Figure 4C**). We observed marked differences between T1 and T2 of the T stage, and N0 and N1 of the N stage (**Supplementary Figures 2**, **3**); however, there was no significant difference in other periods (**Supplementary Figures 4**–**6**). To explore the independent prognosis of risk models and clinicopathological factors, the Cox-independent prognostic analysis of age, the T, M, and N staging, and the RS showed that Pathologic_M and RiskScore were stand-alone prognostic indicators for LUAD (P < 0.05). We next analyzed RSs and various clinical features, including age, sex, tumor stage, T (size or extent of the tumor itself), M (distant metastasis), and N (tumor peripheral lymph node invasion and metastasis). A differential expression heat map of the genes was drawn (**Figure 4D**). Univariate and multivariate results were consistent, indicating that the conclusions were stable and easy to interpret.

### 3.5 Characteristics of immune infiltration in LUSC

**Figure 5:**
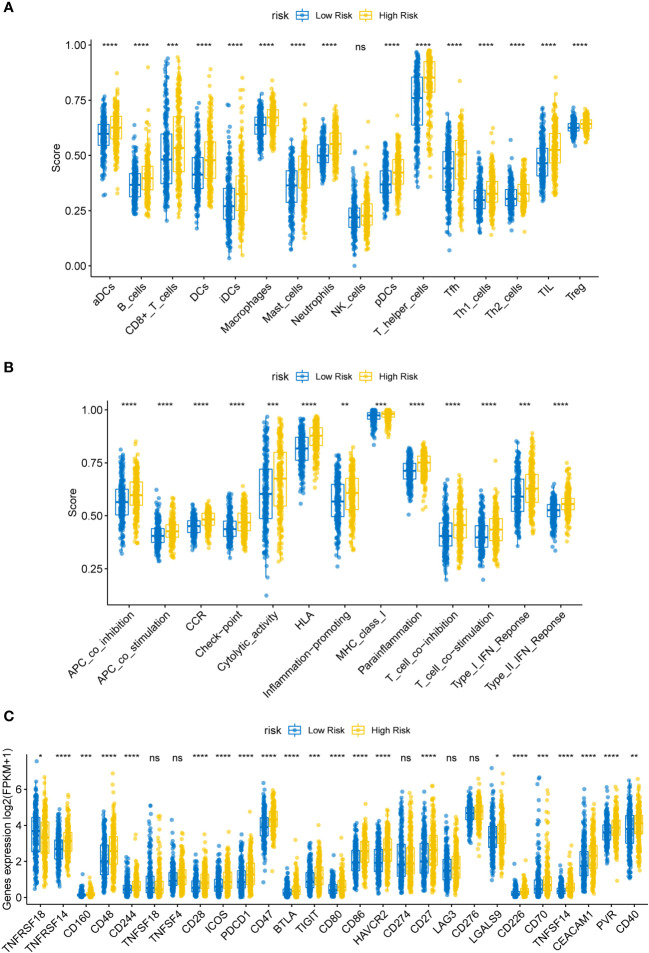
**(A)** Box plot of immune infiltrating cells in the high- (HR) and low-risk (LR) cohorts. The HR cohort was strongly associated with elevated tumor infiltration levels in LUSC (P < 0.05). **(B)** Box plot of tumor infiltrated pathway. **(C)** Box plot of immunoassay sites in the HR and LR cohorts. The levels of the remaining immune checkpoint genes were markedly different between the HR and LR cohorts (P-values < 0.05); ns, not significant. (*P<0.05, **P<0.01, ***P<0.001, ****P<0.0001).

Previous research has revealed a relationship between immune cell invasion and clinical prognosis in cancers, which may be utilized as drug targets to enhance the prognosis of patients (15, 16). Therefore, we quantified the tumor infiltration levels of 29 immune-related gene sets in the HR and LR cohorts. Immune checkpoint inhibitors were reported to be effective potent therapeutic methods against various cancers (17–19); hence, we assessed the levels of key immune checkpoint molecules in LUSC. The HR cohort was markedly correlated with elevated tumor infiltration levels in LUSC (**Figures 5A, B**; all P-values < 0.05); however, the tumor infiltration levels of NK cells showed no significant differences between the HR and LR cohorts. Importantly, the checkpoint scores between the HR and LR cohorts were significantly different. ssGSEA was performed on the samples from the HR and LR cohorts; we observed marked differences in the levels of certain immune cell infiltrates between the HR and LR cohorts. The infiltrating cells included aDCs, B cells, CD8^+^ T cells, DCs, iDCs, macrophages, mast cells, neutrophils, pDCs, T helper cells, Tfh, Th1 cells, Th2 cells, TIL, and Tregs. There were also significant differences in the levels of some immune-linked pathways between the HR and LR cohorts, such as APC_co_inhibition, APC_co_stimulation, CCR, Check-point, Cytolytic_activity, HLA, Inflammation-promoting, MHC_class_I, Parainflammation, T_cell_co-inhibition, T_cell_co-stimulation, Type_I_IFN_Reponse, and Type_II_IFN_Reponse. Immune checkpoints refer to those that inhibit cytotoxic T lymphocyte activation, or cytotoxicity, as well as T lymphocyte (killer T cell) interaction. These findings suggest that the prognostic model is related to the function of antigen-presenting cells (APCs), cytotoxic T cells, immune checkpoints, and major histocompatibility complex (MHC). Thus, the risk model could also be an indicator of tumor immune response in LUSC.

Immune checkpoint molecules for immune function are crucial for TME and immunotherapy (20). To examine the potential association between molecular levels and immune checkpoints, we analyzed the expression of several key immune checkpoint sites, including TNFRSF18, TNFRSF14, CD160, CD48, CD244, TNFSF18, TNFSF4, CD28, ICOS, PD-1 (PDCD1), CD47, BTLA, TIGIT, CD80, CD86, TIM-3 (HAVCR2), PD-L1 (CD274), CD27, LAG3, CD276, LGALS9, CD226, CD70, TNFSF14, CEACAM1, PVR, and CD40. As shown in **Figure 5C**, apart from TNFSF18, TNFSF4, CD274, LAG3, and CD276, the levels of most immune checkpoint genes were markedly different between the HR and LR cohorts (**Figure 5**; all P-values < 0.05).

### 3.6 Cancer stem cell characteristics of the risk model

**Figure 6:**
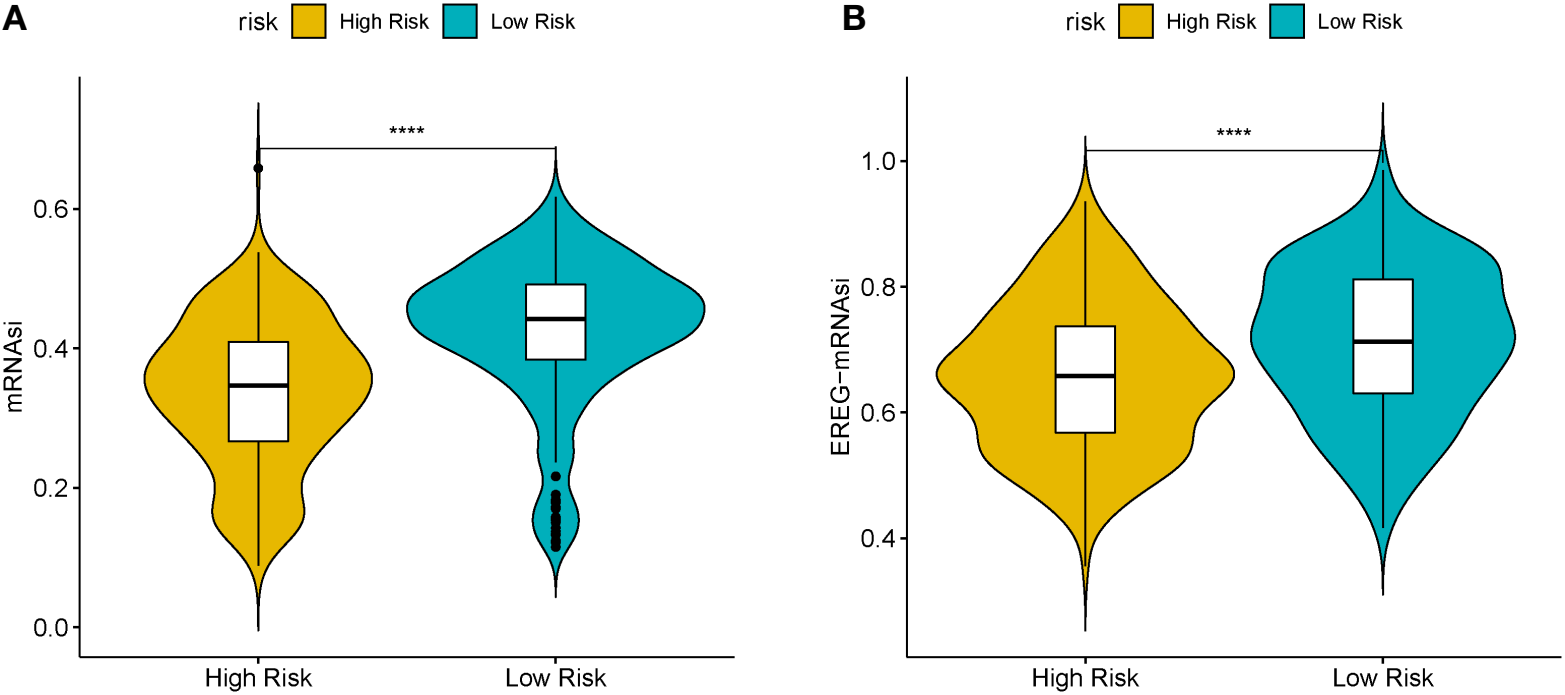
**(A)** Boxplots of mRNAsi in the high- (HR) and low-risk (LR) LUSC patients. **(B)** Boxplots of EREG-mRNAsi in the HR and LR LUSC patients. (*P<0.05, **P<0.01, ***P<0.001, ****P<0.0001).

Cancer stem cells serve essential functions in tumor survival, metastasis, proliferation, and recurrence, owing to their self-renewal ability and production of heterogeneous tumor cells (21). MRNAsi reflects the gene expression characteristics of stem cells. We used mRNAsi as the stemness index to investigate the similarities between cancer and stem cells. The index ranged from 0 to 1; the value of mRNAsi close to 1 indicated enhanced stem cell features of the tumor cells. Thus, the mRNAsi and EREG-mRNAsi of LUSC samples were further computed using the OCLR algorithm and then compared between the HR and LR cohorts. **Figures 6A, B**) shows significant differences in the mRNAsi and EREG-mRNAsi between the two cohorts (P < 0.05).

### 3.7 Establishment of a ceRNA network for LUSC

**Figure 7:**
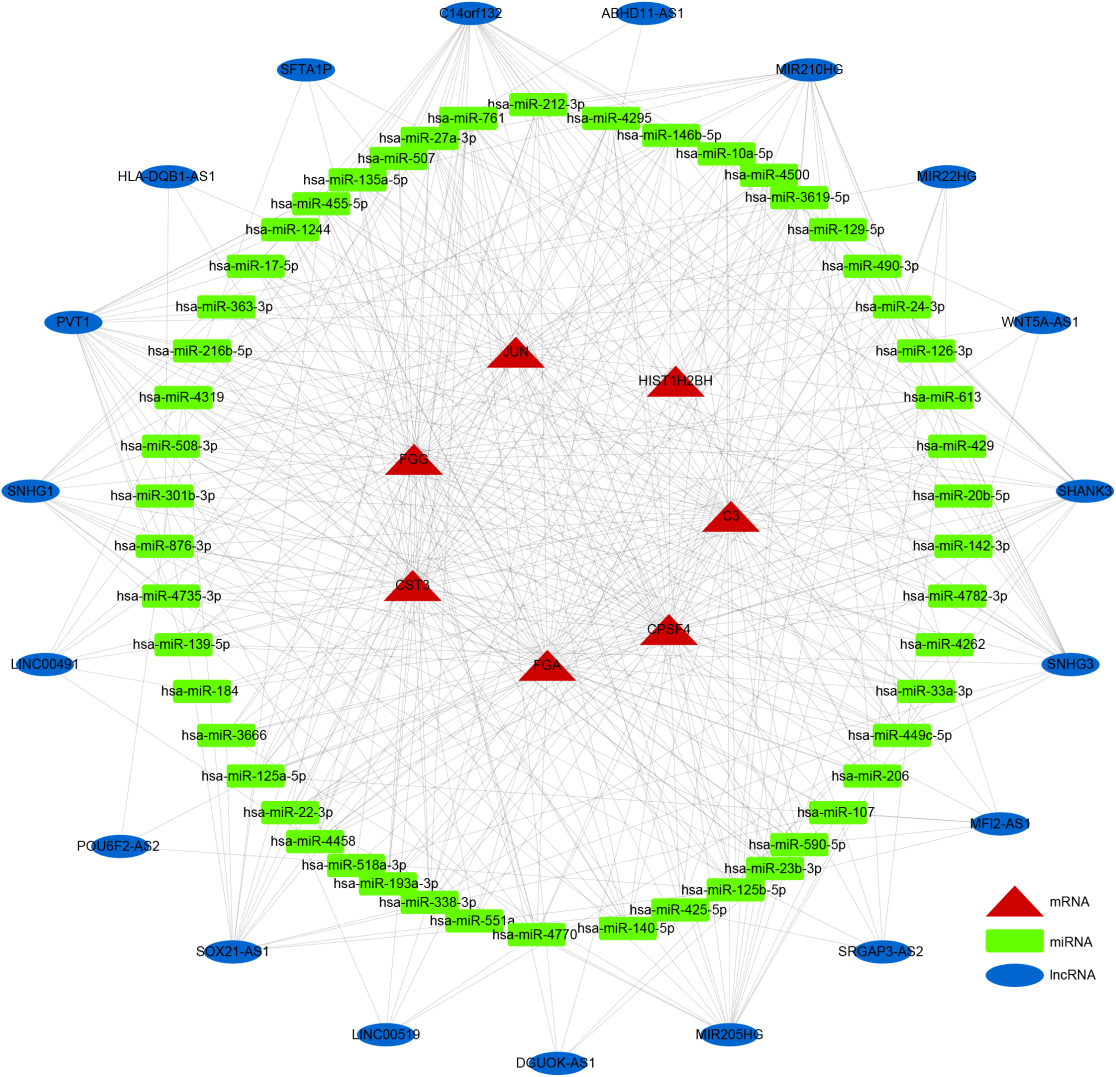
ceRNA network. Rectangles, ellipses, and triangles represent the miRNAs, lncRNAs, and mRNAs of the risk model genes, respectively.

LncRNAs and circRNAs are generally perceived as competing endogenous RNAs (ceRNAs) that bind to miRNAs. ceRNA analysis refers to the analysis of the entire ceRNA regulatory network; usually circRNA-miRNA-mRNA analysis or lncRNA-miRNA-mRNA analysis is perceived as the core of the ceRNA regulatory network. With competitive binding of ceRNAs, such as lncRNA or circRNA with miRNA, the transcription level of the genes regulated by miRNAs will increase. To further elucidate the potential regulatory mechanism of these seven prognostic DEGs in LUSC prognosis, we generated a ceRNA network using the DE-lncRNAs and prognostic DEGs. The target miRNAs of DE-lncRNAs were predicted using the miRcode database, and the target miRNAs of prognostic DEGs were predicted using the miRanda database. A ceRNA network containing 19 lncRNAs, 50 miRNAs, and 7 prognostic DEGs demonstrated the molecular mechanism of LUSC-specific DEGs in LUSC prognosis (**Figure 7**).

### 3.8 FGG and Clinical Parameters in patients with LUSC

**Figure 8:**
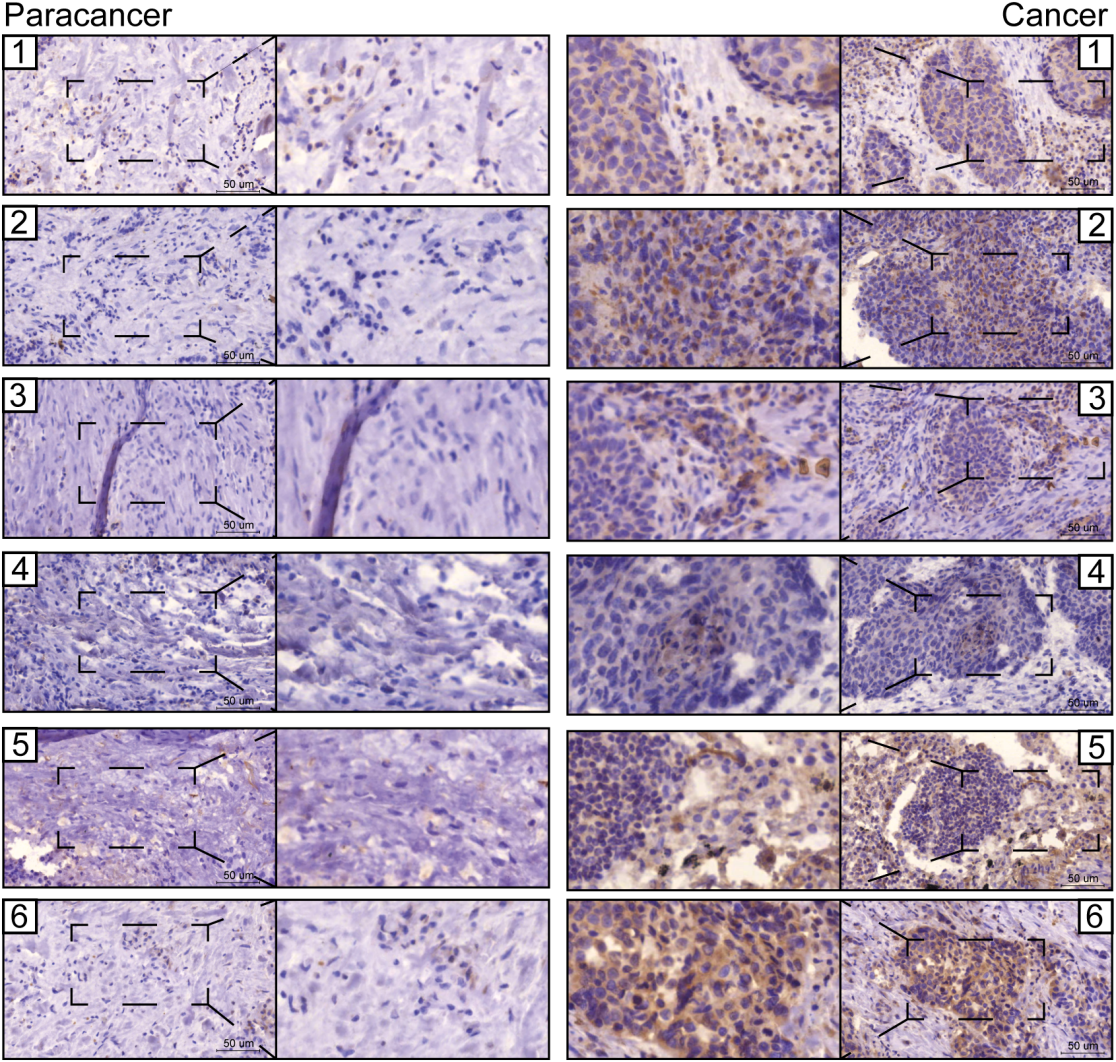
Representative images from immunohistochemical staining of FGG in lung cancers (n = 6) and normal tissues (n = 6). Scale bars: 100 μm and 50 μm.

The prognostic values of FGG, C3, FGA, JUN, CST3, CPSF4, and HIST1H2BH7 genes in LUSC in the TCGA database suggest that they may play a role as key risk factors in tumors (**Supplementary Figure 7**). The expressions of seven prognostic genes in human LUSC cell lines NCI-H520 and LTEP-s were detected using q-PCR; FGG was significantly highly expressed in both LUSC cell lines (**Supplementary Figure 8**). We also examined the expression of FGG in surgically collected, paired, LUSC samples, and adjacent normal tissues from 6 patients. Remarkably, all LUSC specimens had markedly increased FGG protein levels compared with matched adjacent normal tissues (**Figure 8**). Our clinical observations reveal that FGG is significantly hyper-expressive in LUSC patient samples, further demonstrating the clinical value of FGG in LUSC.

### 3.9 FGG correlates with tumor progression, immune infiltration, and stem index in LUSC

**Figure 9:**
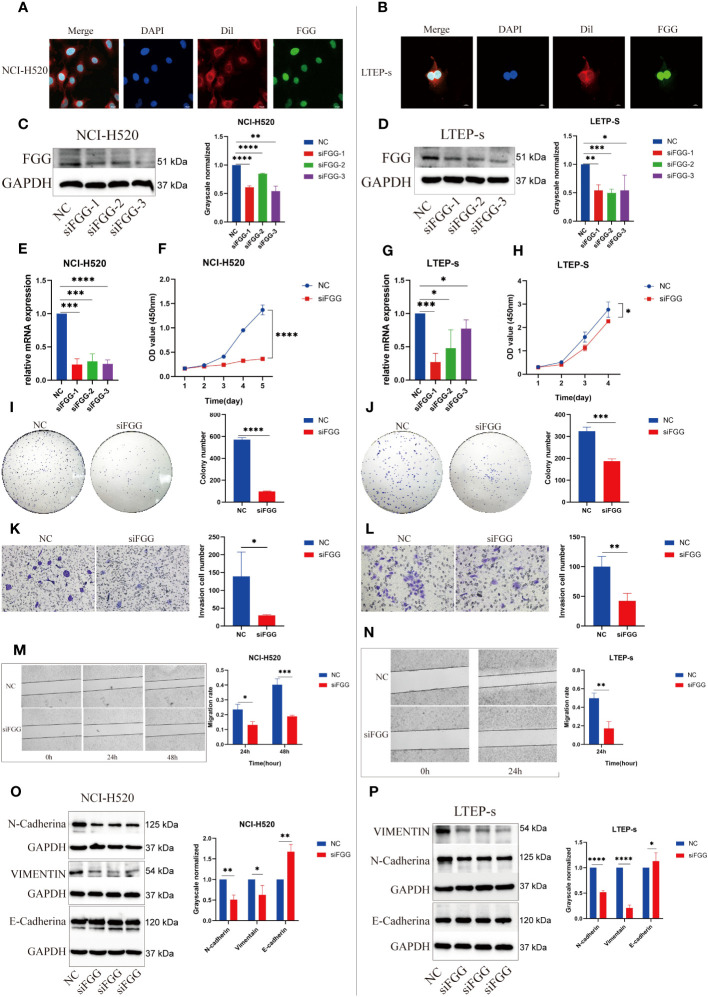
Expression locations of FGG detected using immunofluorescence in **(A)** NCI-H520 and **(B)** LTEP-s. FGG knockdown was determined using western blotting in **(C)** NCI-H520 and **(D)** LTEP-s cells. FGG knockdown was determined using Q-PCR in **(E)** NCI-H520 and **(G)** LTEP-s cells. CCK-8 assay was used to detect the proliferation of **(F)** NCI-H520 and **(H)** LTEP-s cells Viability line graph **(I)** NCI-H520 and **(J)** LTEP-s cell colony formation result. The result of the invasion of **(K)** NCI-H520 and **(L)** LTEP-s cells. The results of Wound Healing and migration of **(M)** NCI-H520 cells and **(N)** LTEP-s cells. Western blotting assay showing EMT markers N-cadherin, Vimentin, and E-cadherin expression following FGG knockdown in **(O)** NCI-H520 and **(P)** LTEP-s cells. The significant differences were analyzed using GraphPad Prism *t*-test, n=3 (*P<0.05, **P<0.01, ***P<0.001, ****P<0.0001).

Immunofluorescence showed that FGG was expressed in the nucleus of LUSC (**Figures 9A, B**). To demonstrate the biological function of FGG in LUSC cells, NCI-H520 (**Figures 9C, E**) and LTEP-s cell lines (**Figures 9D, G**) with FGG knockdown were successfully constructed.

**Figure 10:**
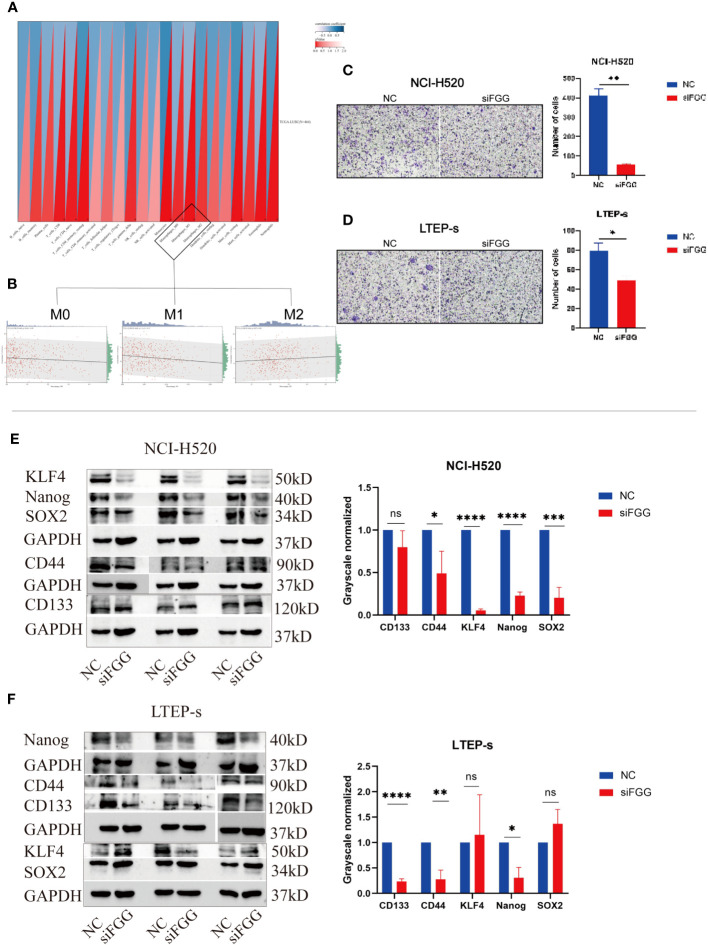
**(A)** Pearson’s correlation coefficient of FGG with 22 immune cell infiltration scores in LUSC was calculated using the corr.test function of the R package psych (version 2.1.6), and 10 significantly correlated immune infiltration scores, including macrophages, were identified, **(B)** for further individual correlations plotted for FGG with M0, M1, and M2 macrophages, respectively (p=0.04, r=0.09, p=1.1e-03, r=0.15, p=0.07, r=0.09). Tranwell shows the infiltration of THP-1of **(C)** NCI-H520 cells and **(D)** LTEP-s cells. Western blotting assay showing the expression of stemness marker genes SOX2, Nanog, CD133, CD44, KLF4 following FGG knockdown in **(E)** NCI-H520 and **(F)** LTEP-s cells. "ns" No Significant.

Our results suggest that FGG can affect the tumor process of LUSC cells, as shown by the proliferation (**Figures 9F, H**), cloning (**Figures 9I, J**), invasion (**Figures 9K, L**), and migration (**Figures 9M, N**) of NCI-H520 and LTEP-s being significantly inhibited following FGG knockdown. In addition, the result of western blot showed that the expression of E-cadherin was increased while that of N-cadherin and VIMENTIN were decreased following FGG knockdown, which also corresponded to the inhibition of migration and invasion (**Figures 9O, P**). Subsequently, we evaluated the scores of 22 kinds of tumor immune cell infiltration in LUSC patients according to the expression of FGG and found that FGG was significantly correlated with 10 kinds of immune cell infiltration, including M1 macrophages (**Figures 10A, B**). *In vitro* Transwell experiments showed that the ability of NCI-H520 (**Figure 10C**) and LTEP-s cell lines (**Figure 10D**) with low FGG to recruit M1 mononuclear macrophages was significantly down-regulated. After FGG knockdown, KLF4, Nanog, CD44, and SOX2 in NCI-H520 cells were significantly decreased, while CD133 showed no significant changes (**Figures 10E, F**). After FGG knockdown, KLF4, Nanog, CD44, and SOX2 in NCI-H520 cells were significantly decreased, while CD133 showed no significant changes (**Figure 10E**). After FGG downregulation, the expressions of CD44 and CD133 in LTEP-s cells were significantly decreased, while no significant changes in KLF4, Nanog, and SOX2 were observed (**Figure 10F**). These results indicate that FGG affected the tumor progression, immune infiltration, and stem index of LUSC cells.

## 4 Discussion

LUSC is a subtype of NSCLC and accounts for nearly 40% of all lung cancers. Early detection and the prognostic assessment of LUSC remain challenging, hence the poor 5-year survival rate of patients (22). Recent studies have improved the prognosis prediction for LUSC patients, focusing on biomarkers. For example, Shi et al. investigated DNA methylation profiling and proposed potential diagnostic biomarkers for LUSC (23). Chen et al. investigated the roles of IRGs in the deterioration of lung cancer and indicated the distinction between LUAD and LUSC from the perspective of the immune response (24). Liao et al. identified biomarkers with cancer stem cell characteristics in LUSC (14). To date, the prognostic gene signatures for prognostic prediction of LUSC are scarce and warrant further investigations. Several studies have proposed prognostic markers for survival prediction in patients with LUSC. Zhang et al. suggested that IRGPI could be used as a prognostic marker (25), while Li et al. constructed an mRNA signature to predict the outcomes of patients with LUSC (26). Liu et al. have identified an miRNA signature with potential clinical implications in the outcome prediction of LUSC (27). Indeed, several lncRNAs, such as VPS9D1-AS1 and MALAT-1, are correlated with the survival of LUSC patients (28, 29). Huang et al. reported a nine-long non-coding RNA signature for prognosis prediction of patients with LUSC (7). However, no prognostic indicators of LUSC have been established based on tumor progression, immune infiltration, and stem index analysis.

Recent studies have found that LUSC differs from LUAD in terms of genomic, epigenetic, CSC stemness, and TME characteristics. According to previous research, CSCs may lead to cancer recurrence and drug resistance (30, 31). The TME is a mutually adaptive environment in which tumor cells escape immunological surveillance. Tumor progression involves crosstalk between CSCs and the TME (32, 33), such as the induction of CSCs in EMT (34) and the interaction of angiogenesis and components of the TME (35). Herein, we adopted a comprehensive perspective of cancer biology based on tumor progression, TME, and CSC index for a better understanding of LUSC as an independent NSCLC from different dimensions. We recognized the importance of the particularly expressed genes in LUSC based on the TCGA database and DEGs in HR and LR cohorts; from this, we recognized the functions of independent genes as potential predictors of tumor invasion, metastasis, tumor stem cell characteristics, and immune cell infiltration. Seven prognostic genes were varied in LUSC and were associated with the TNM stage and prognosis; these genes were FGG, C3, FGA, JUN, CST3, CPSF4, and HIST1H2BH. FGG, C3, FGA, JUN, and CST3 were associated with poor outcomes in LUSC patients, whereas CPSF4 and HIST1H2BH served as positive prognostic markers in LUSC patients.

In terms of clinicopathological features, the seven-gene biomarkers showed differences in tumor metastasis and invasion, and the significant differences between T1 and T2 of the T stage and N0 and N1 of the N stage suggested that the modification occurred during the early stage of tumor disease; however, the factors of dabbling were limited, such as the lack of the status of smoking status, driver factors, ORR of the various chemotherapy, and immune checkpoint blockade subgroups. Kaplan–Meier analysis showed that LUSC patients in the LR group exhibited significantly higher OS than those in the HR group, while ROC curve analysis results showed that this gene profile could effectively predict the OS of LUSC. Subsequently, our independent prognostic value analysis showed that protective genes were highly expressed in the low-risk group, while the risk genes were highly expressed in the high-risk group, indicating stable results. Moreover, the ROC curve showed that RS could be used as an independent prognostic factor effectively predicting LUSC outcomes. We also analyzed the relationships between HR and LR cohorts, immunoinfiltrating cells, and immune pathways, and showed that HR patients exhibited significantly elevated levels of tumor cell immune infiltration and that the molecular expression of immune checkpoint genes significantly differed between HR and LR patients.

Next, we analyzed the stem cell characteristics of the model and showed that mRNA was associated with prognosis and relevance; significant differences were noted in mRNAsi and EREG-mRNAsi between HR and LR patients, providing new insights into the clinical features, immune response, and TME of tumors based on the dry index. Finally, we constructed a ceRNA network containing 19 lncRNAs, 50 miRNAs, and 7 prognostic DEGs, demonstrating the prognostic value of novel biomarkers for Lusc-specific DEGs.

The prediction of the risk prognostic model constructed can potentially provide more reliable theoretical support for clinical application. However, bioinformatics is only a short practical perspective to this goal; therefore, we conducted specific molecular studies on prognostic genes. Based on the risk model constructed above, combined with RT-qPCR assay and survival analysis of the TCGA database, we screened LUSC-specific prognostic genes and found that FGG was closely correlated with LUSC results in univariate Cox analysis (P=0.000427708), and mRNA levels of FGG were stably expressed in NCI-H520 and LTEP-s cells and significantly up-regulated compared with normal airway epithelial cells. Therefore, their roles in tumor progression, immunoinfiltration, and dry characteristics were further analyzed.

FGG is the γ-chain of fibrinogen, a large, complex glycoprotein with a total molecular mass of approximately 340 kDa, comprising three pairs of polypeptide chains: Aα (encoded by the FGA gene), Bβ (FGB), and γ (36). FGG has a conserved globular domain, γC, at the COOH terminus, which is a major integrin binding site for fibrinogen. Yokoyama et al. found that the C-terminal region of FGG, as the primary integrin binding site of fibrinogen, participated in the process of thrombosis, angiogenesis, and inflammation (37, 38). Nobuaki Akakura et al. found that isolated γC and its mutant γC399tr induce endothelial cell apoptosis, and recombinant soluble γC399tr inhibited tumor growth, intratumoral vascular development, and metastasis *in vivo* (39). Previous studies have shown that fibrinogenemia, as a prognostic factor (40–42), is often observed in patients with malignant tumors and is closely related to tumor invasion, metastasis (43–45), angiogenesis (46), and tumor growth processes (47); further, its degradation products with carcinogenesis have been reported in tumors (41). However, Nagata et al. found that frameshift mutations in FGG led to hypofibrinemia, indicating that FGG was involved in the regulation of fibrinogen secretion (48). In addition, FGG inhibits platelet adhesion to fibrinogen by interacting with hepatitis B splicing protein (49). Dysregulation of FGG has also been reported in many malignant tumor types, such as liver cancer (50), stomach cancer (40), and prostate cancer (51), underscoring its potential relevance as a tumor marker. FGG is an important adverse prognostic factor for gastric cancer (35). Another study showed that serum FGG levels predicted the progression of prostate cancer (51). Additionally, FGG is thought to distinguish cancer from normal sera as a potential tumor marker in pancreatic cancer (52). Additional data show the possibility of urine FGG levels as a potential diagnostic marker for NSCLC (53). These findings suggest that FGG could hold diagnostic, prognostic, and therapeutic implications in cancer.

Our bioinformatics modeling demonstrated that FGG as a risk prognosticator is of significant research value in LUSC, and the results of subsequent *in vitro* experiments are consistent with reports of abnormal expression of FGG mRNA in various cancers. Knockdown of FGG caused functional changes in LUSC tumor progression at the tumor cell level, significantly inhibited the proliferation and clonogenesis ability of NCI-H520 and LTEP-s cells, and blocked the cell cycle in the S phase (**Supplementary Figure 9**). It also inhibited the invasion and migration ability of tumor cells, by reducing the EMT process and promoting the early apoptosis of tumor cells. In terms of dry characteristics, FGG down-regulation decreased the expressions of KLF4, Nanog, CD44, and SOX2 in NCI-H520 cells, and the expressions of CD44, Nanog, and CD133 in LTEP-s cells. In terms of immune cell infiltration, the expression of FGG in LUSC tissues was significantly correlated with M0 and M1 type macrophages, while knockdown of FGG in LUSC cells significantly affected the degree of immune infiltration of M1 type macrophages (**Supplementary Figure 10**) formed by polarization of THP-1 cells, suggesting that FGG plays a specific role in the immune infiltration of LUSC.

In summary, our study successfully constructed a LUSC-specific DEGs based risk and prognosis model and verified the reliability of the risk model from the data model. According to the prognostic risk factors, including tumor invasion, metastasis, survival, immune infiltration, and tumor stem cell-related genes, DEGs in LUSC were used to determine associations between functional genes and tumor progression, immune invasion, and dry index. However, this prognostic model has some limitations, such as the relatively simple database and limited factors analyzed (such as lack of smoking status, drivers, ORRs of various chemotherapy treatments, and subsets of immune checkpoint blocking). Subsequently, *in vitro* studies of the LUSC-specific prognostic marker FGG will provide deeper insights into LUSC. As a risk factor in this prognostic model, FGG significantly inhibited the progression of LUSC tumor cells after knockdown and reduced the expression of dry marker genes and the infiltration level of M1 type macrophages, suggesting that FGG is a potential biomarker for independent poor prognosis of LUSC to identify LUSC patients with poor clinical outcomes and that it may play specific roles in dry maintenance and immune infiltration. However, the specific mechanism underlying the changes in tumor progression warrants further study.

## 5 Conclusion

This study established a seven-gene profile (FGG, C3, FGA, JUN, CST3, CPSF4, and HIST1H2BH) prognostic stratification system demonstrated in LUSC based on Tumor Progression, Immune Infiltration, and Stem Index. *In vitro* experiments confirmed that DEGs FGG could be independent prognostic biomarkers of LUSC promoting cell proliferation, migration, invasion, THP-1 cell infiltration, and stem cell maintenance.

## Data availability statement

The original contributions presented in the study are included in the article/**Supplementary Material**. Further inquiries can be directed to the corresponding authors.

## Ethics statement

The current study was approved by the institutional ethics review board of Affiliated Hospital of Inner Mongolia Medical University, Hohhot, Inner Mongolia Autonomous Region, China (NO.KY(2021021). The studies were conducted in accordance with the local legislation and institutional requirements. The participants provided their written informed consent to participate in this study.

## Author contributions

RW contributed to the study’s conception, design, and manuscript writing. RM conducted the *in vitro* experiment. XD performed the bioinformatics analysis. JZ and KL generated all the Figures and Tables. LY and MZ contributed to the manuscript editing. RW was responsible for funding acquisition. CW and PL reviewed and approved the manuscript. All authors contributed to the article and approved the submitted version.
