# Supplementary figures and images for: Identification of specific prognostic markers for lung squamous cell carcinoma based on tumor progression, immune infiltration, and stem index
Source: Front Immunol. 2023 Sep 29;14:1236444. doi: 10.3389/fimmu.2023.1236444 (PMC10570622; doi:10.3389/fimmu.2023.1236444)

(a)

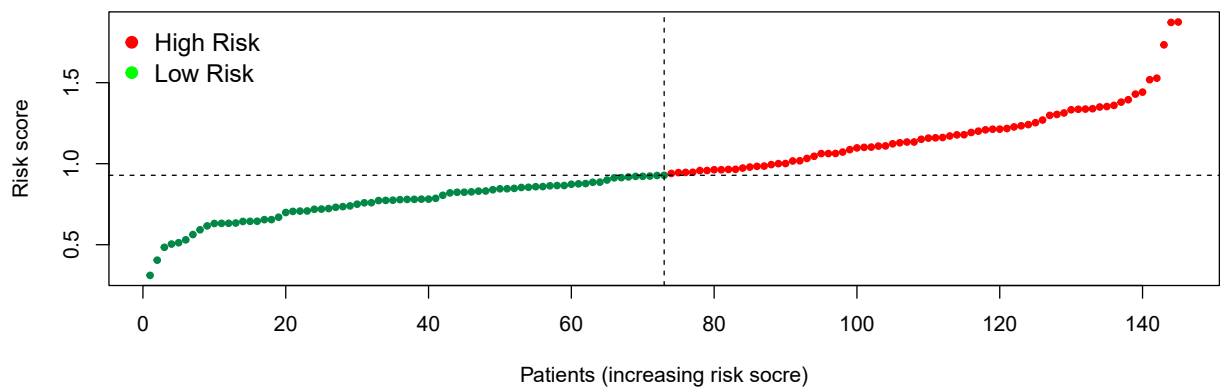

(b)

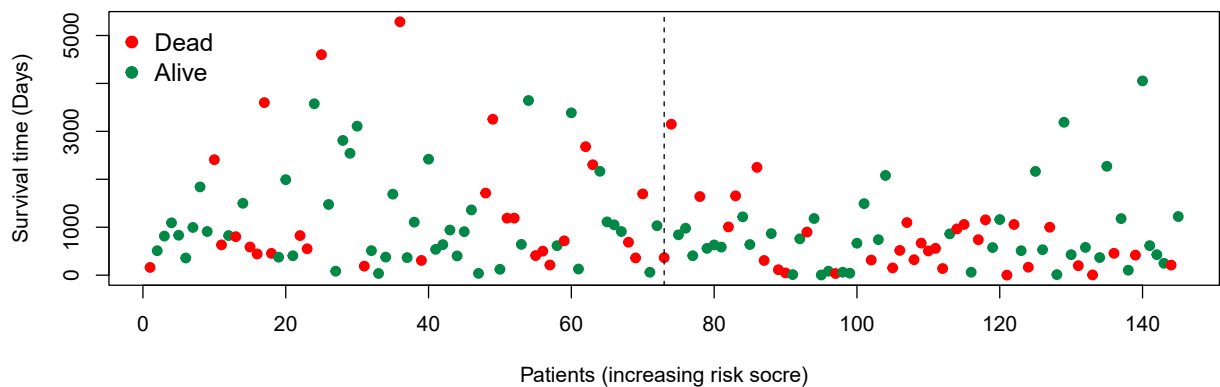

Supplement: Supplementary file 1 [file DataSheet_1.pdf]

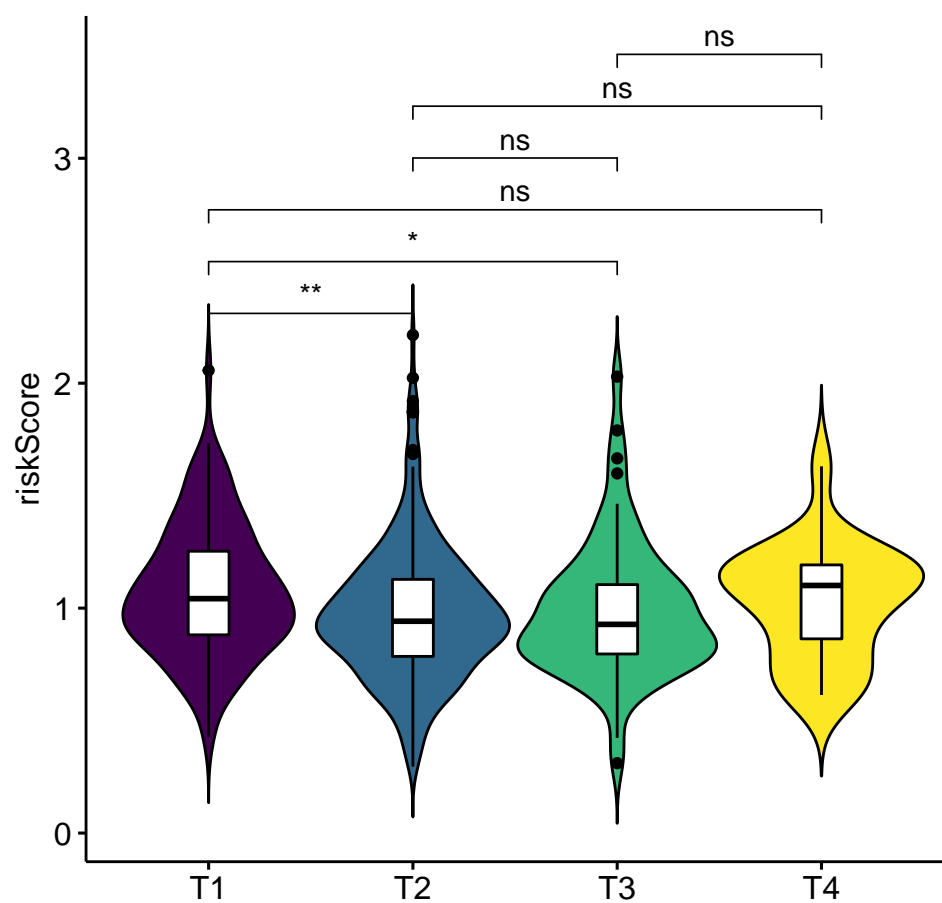

Supplement: Supplementary file 2 [file DataSheet_2.pdf]

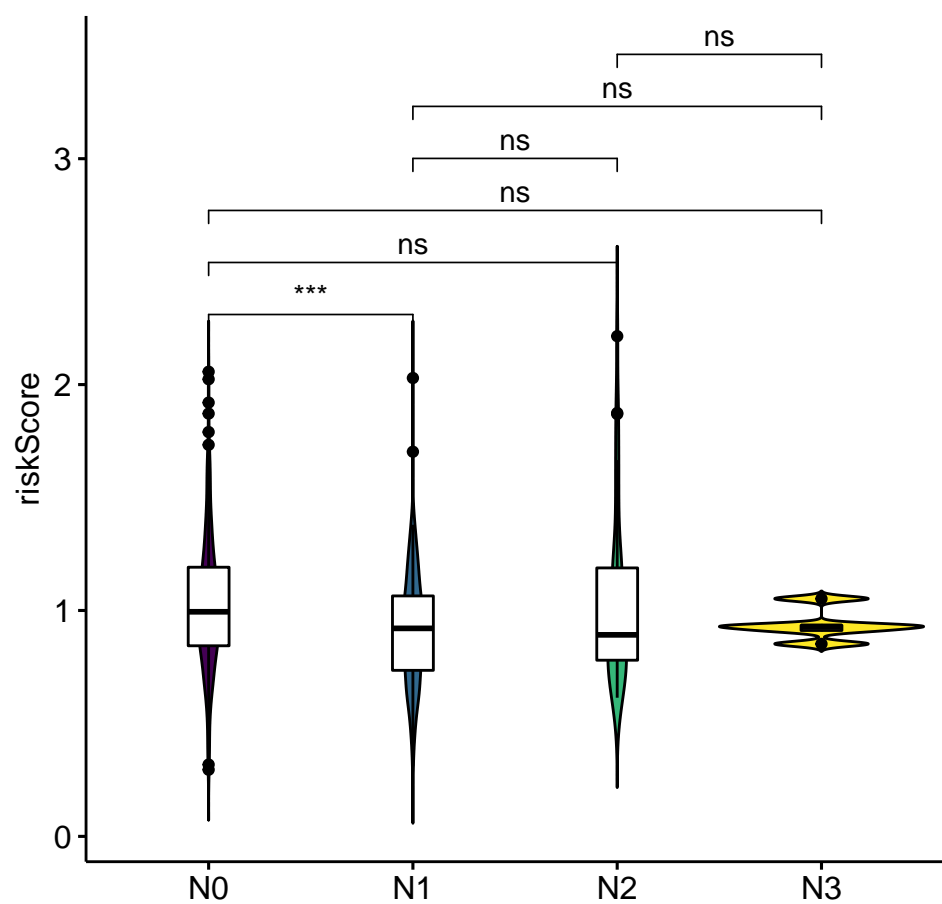

Supplement: Supplementary file 3 [file DataSheet_3.pdf]

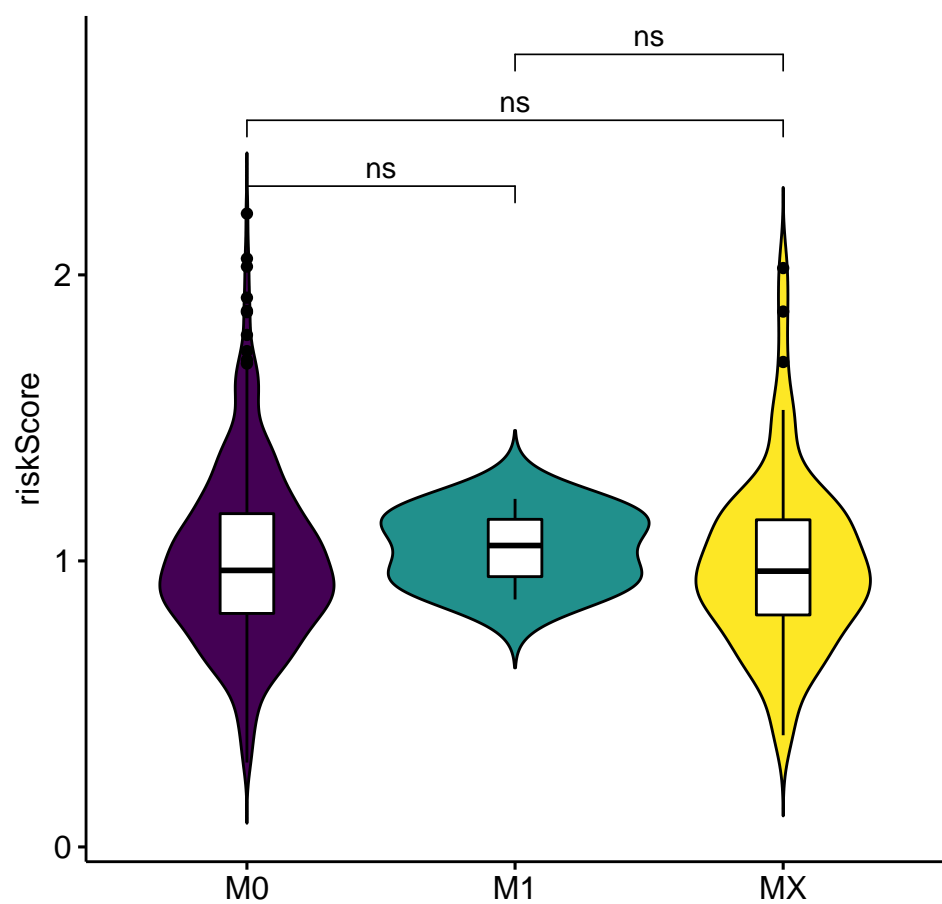

Supplement: Supplementary file 4 [file DataSheet_4.pdf]

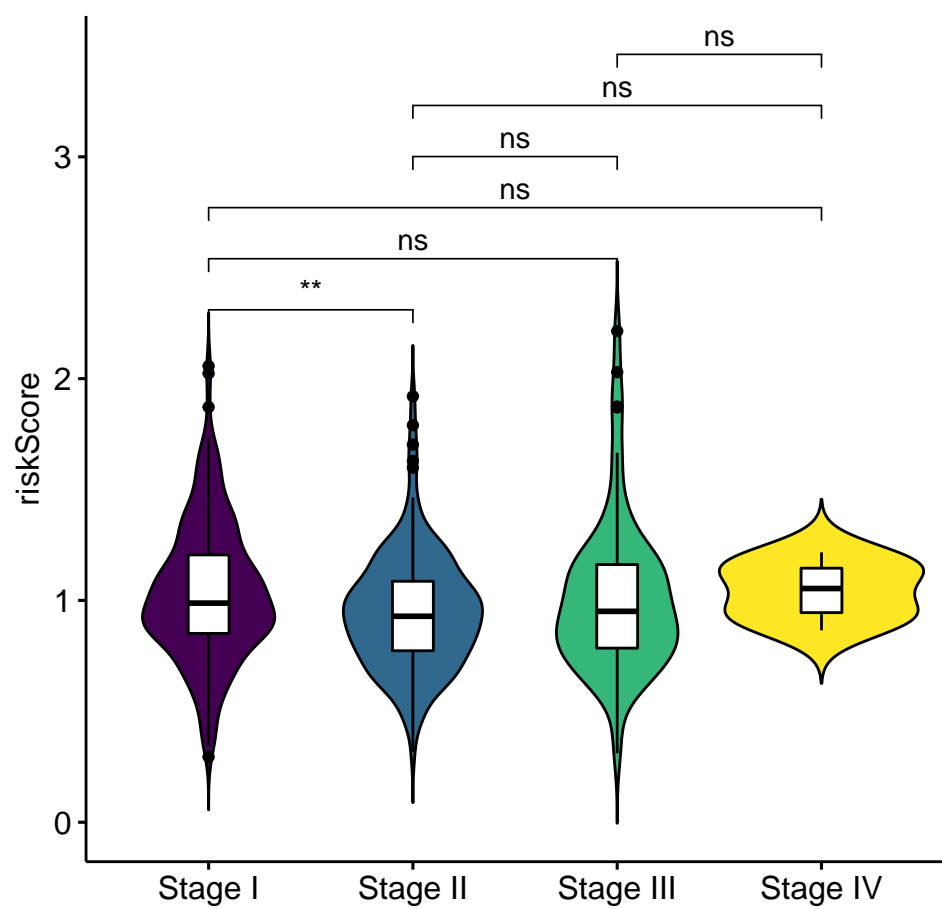

Supplement: Supplementary file 5 [file DataSheet_5.pdf]

age  Age >60  Age <=60

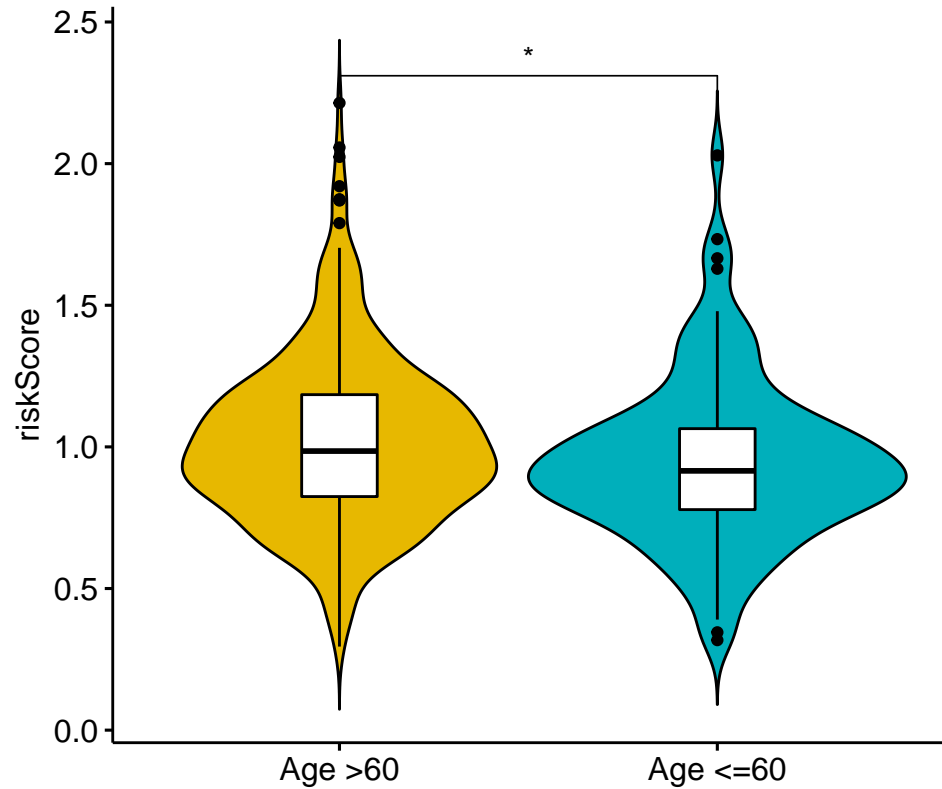

Supplement: Supplementary file 6 [file DataSheet_6.pdf]

FGG

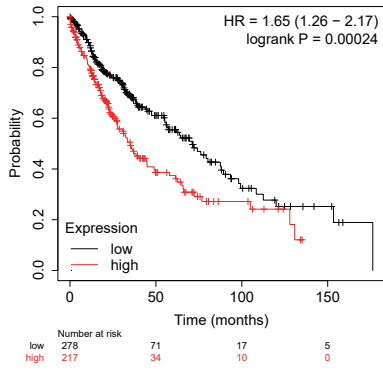

FGA

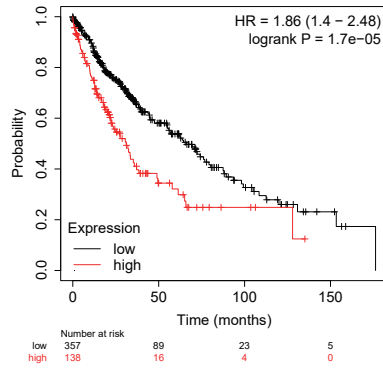

C3

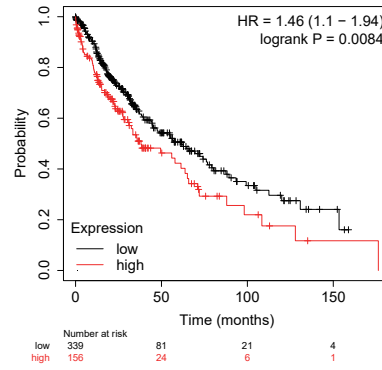

HIST1H2BH

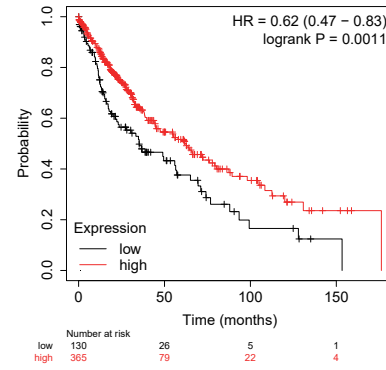

JUN

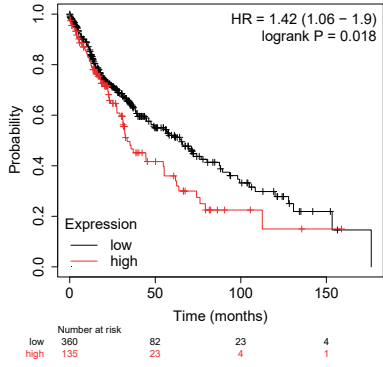

CPSF4

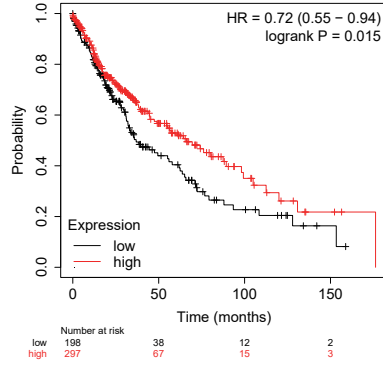

CST3

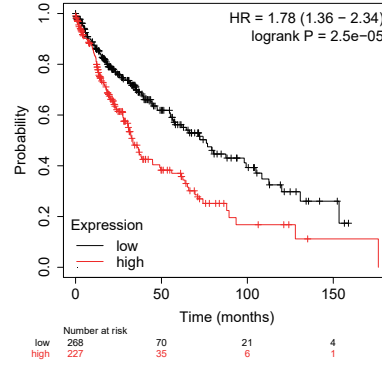

Supplement: Supplementary file 7 [file DataSheet_7.pdf]

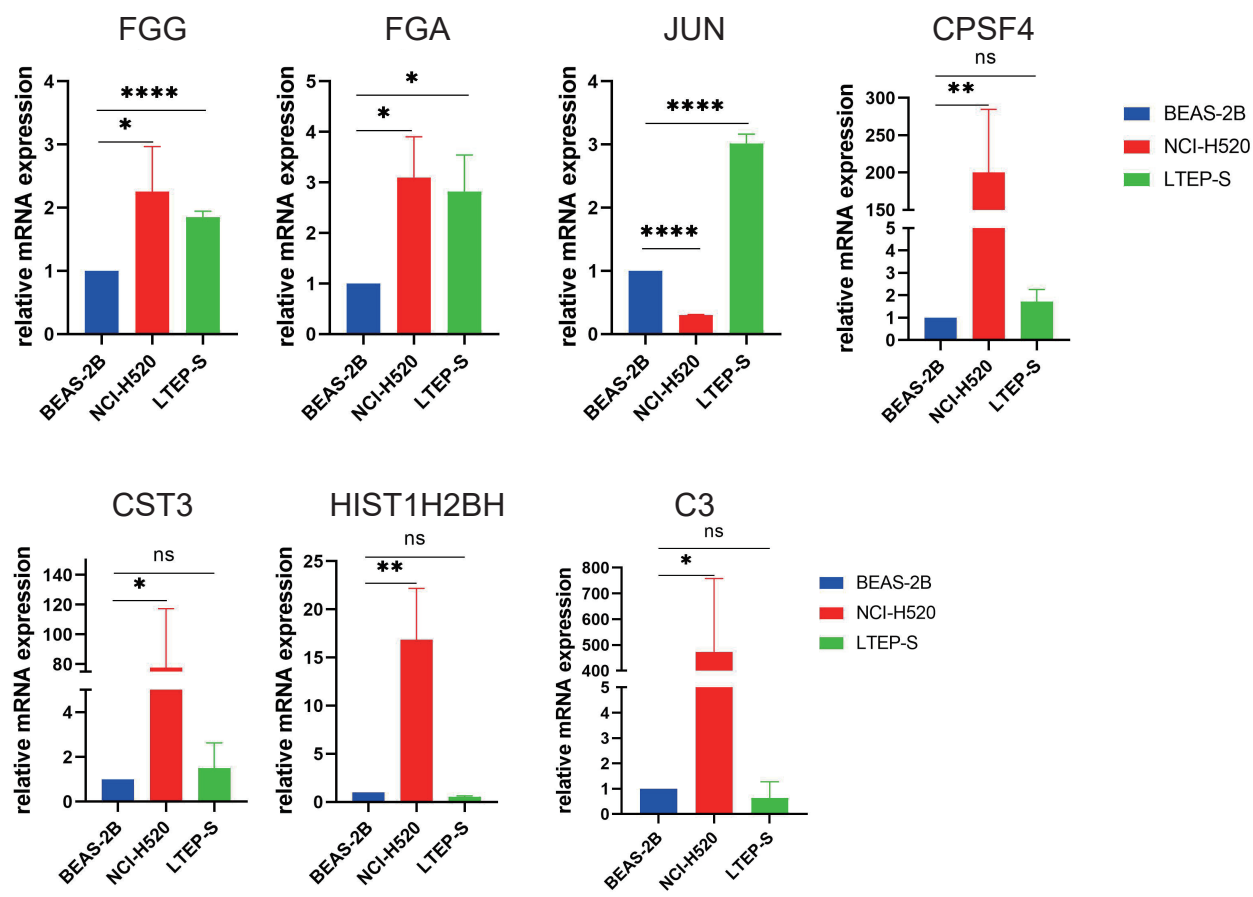

Supplement: Supplementary file 8 [file DataSheet_8.pdf]

a

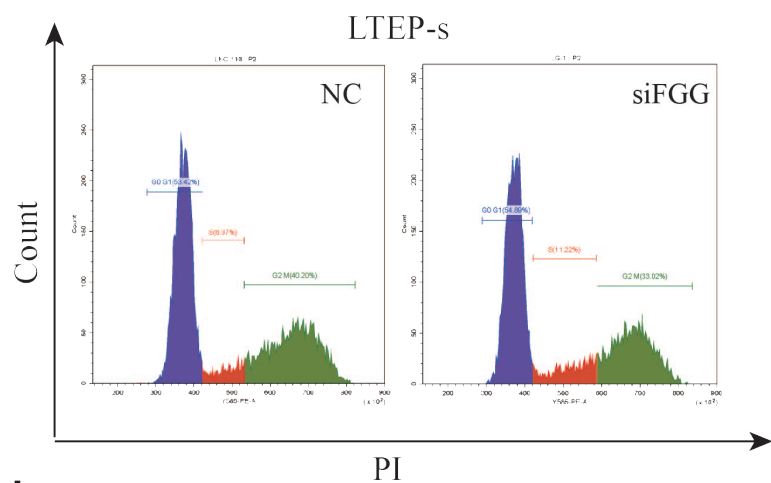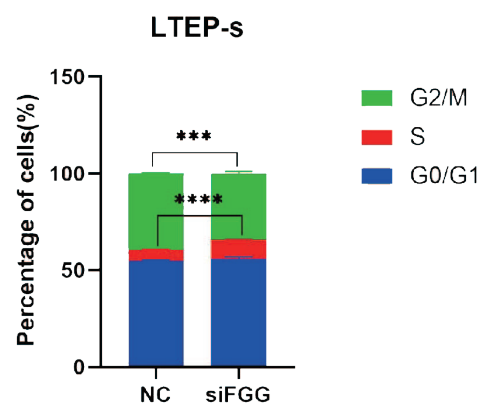

b

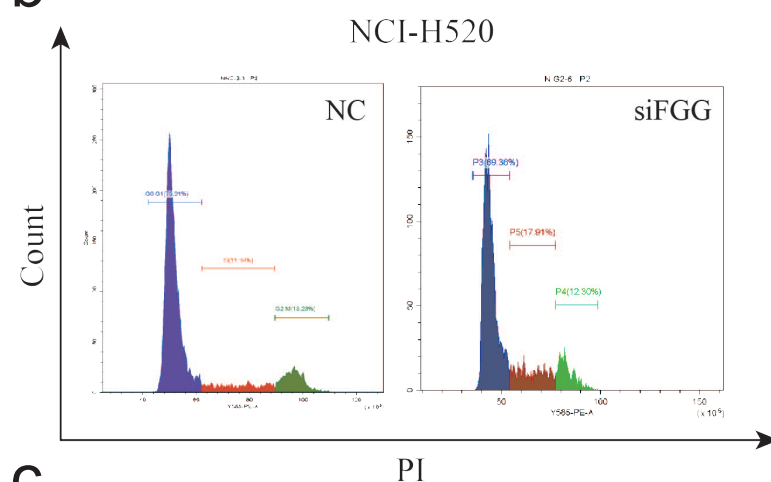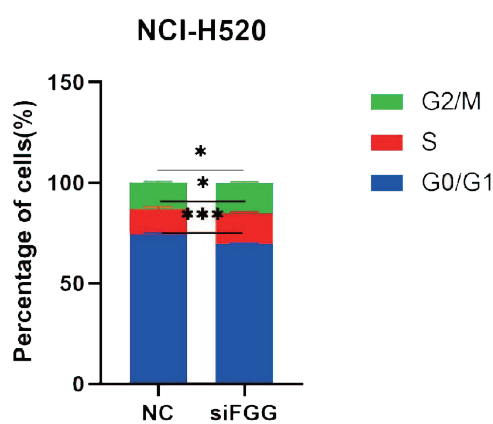

c

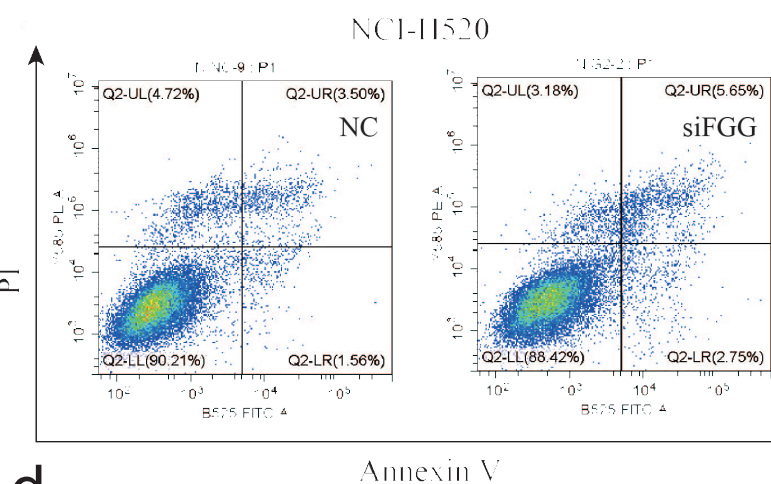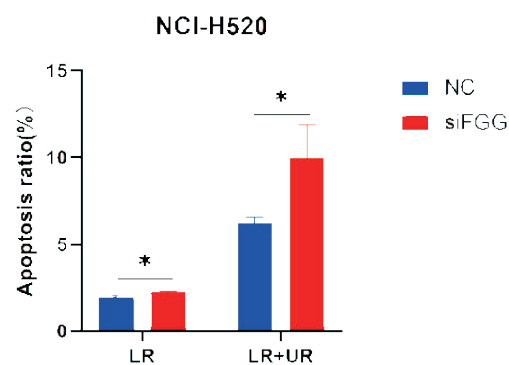

d

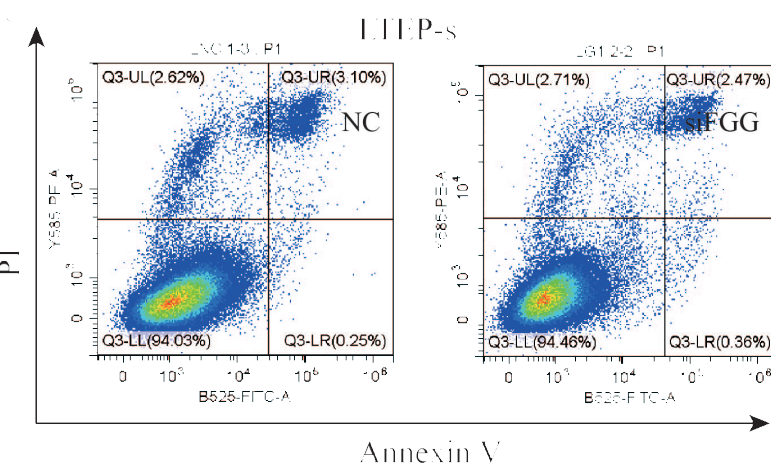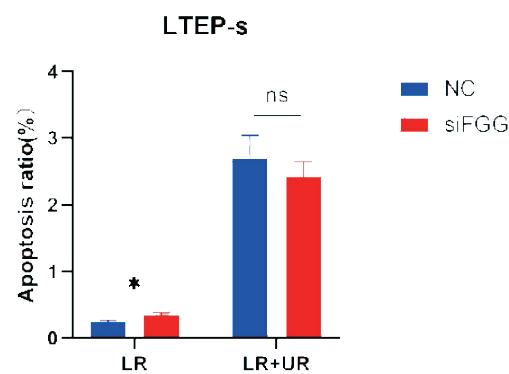

Supplement: Supplementary file 9 [file DataSheet_9.pdf]

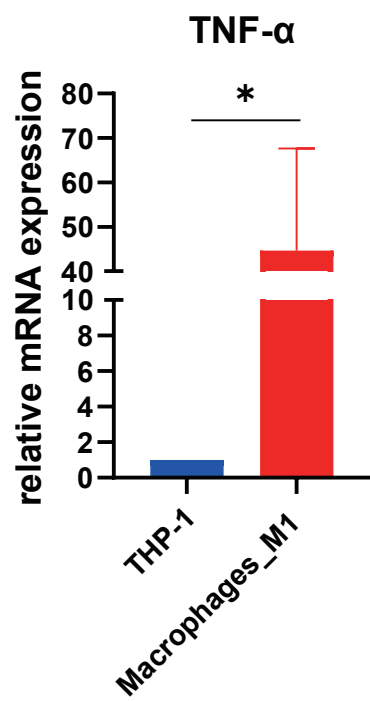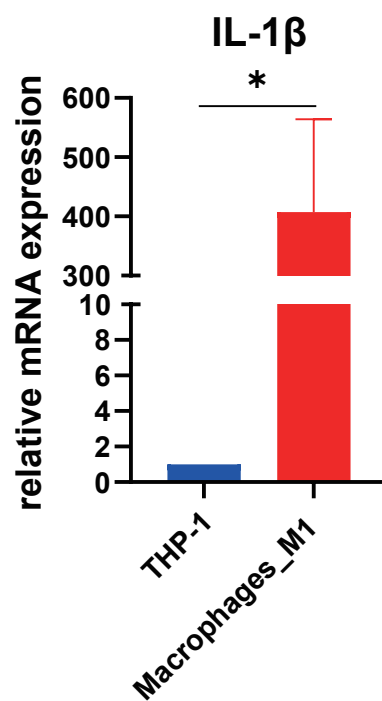

Supplement: Supplementary file 10 [file DataSheet_10.pdf]

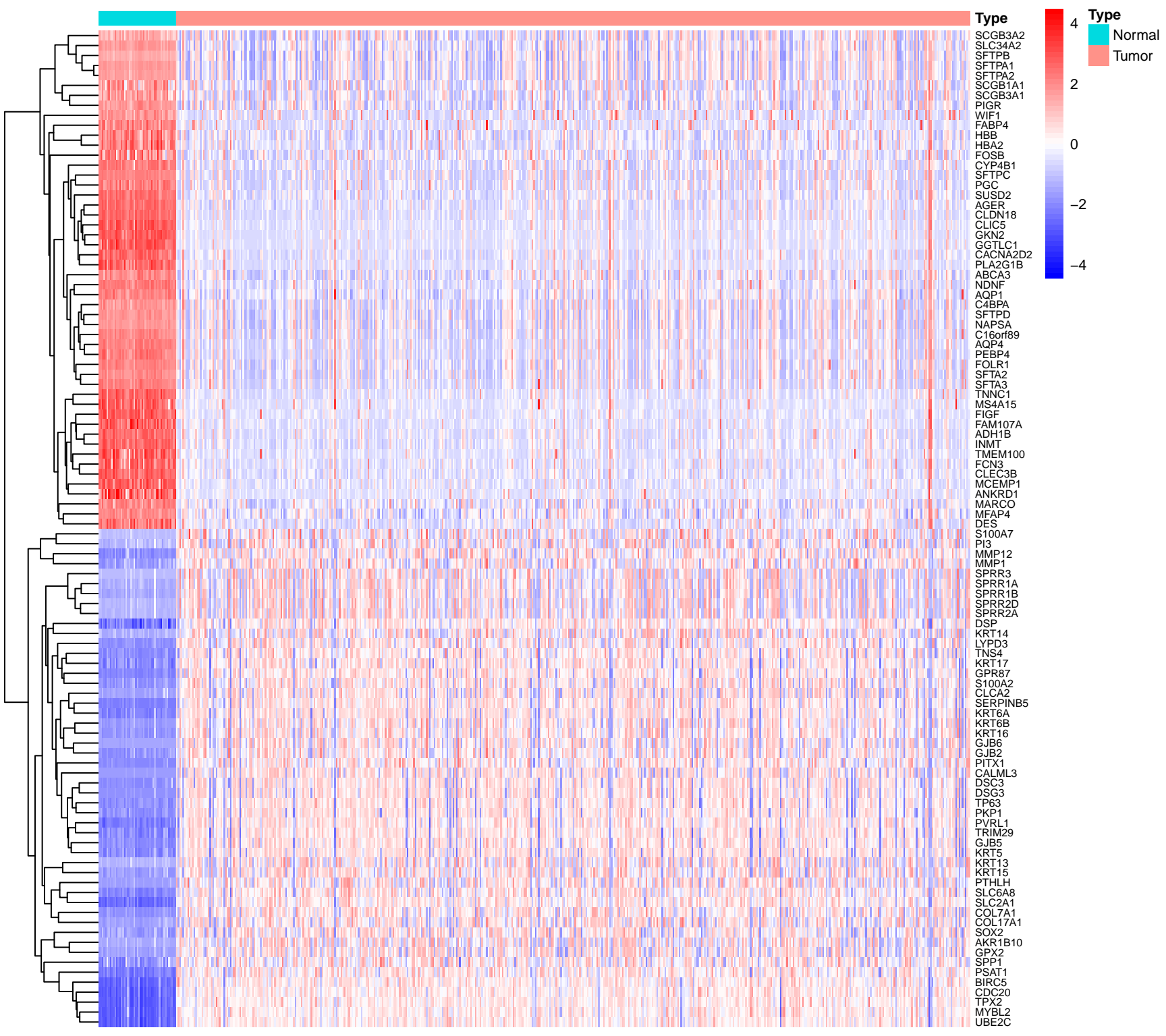

Supplement: Supplementary file 11 [file DataSheet_11.pdf]

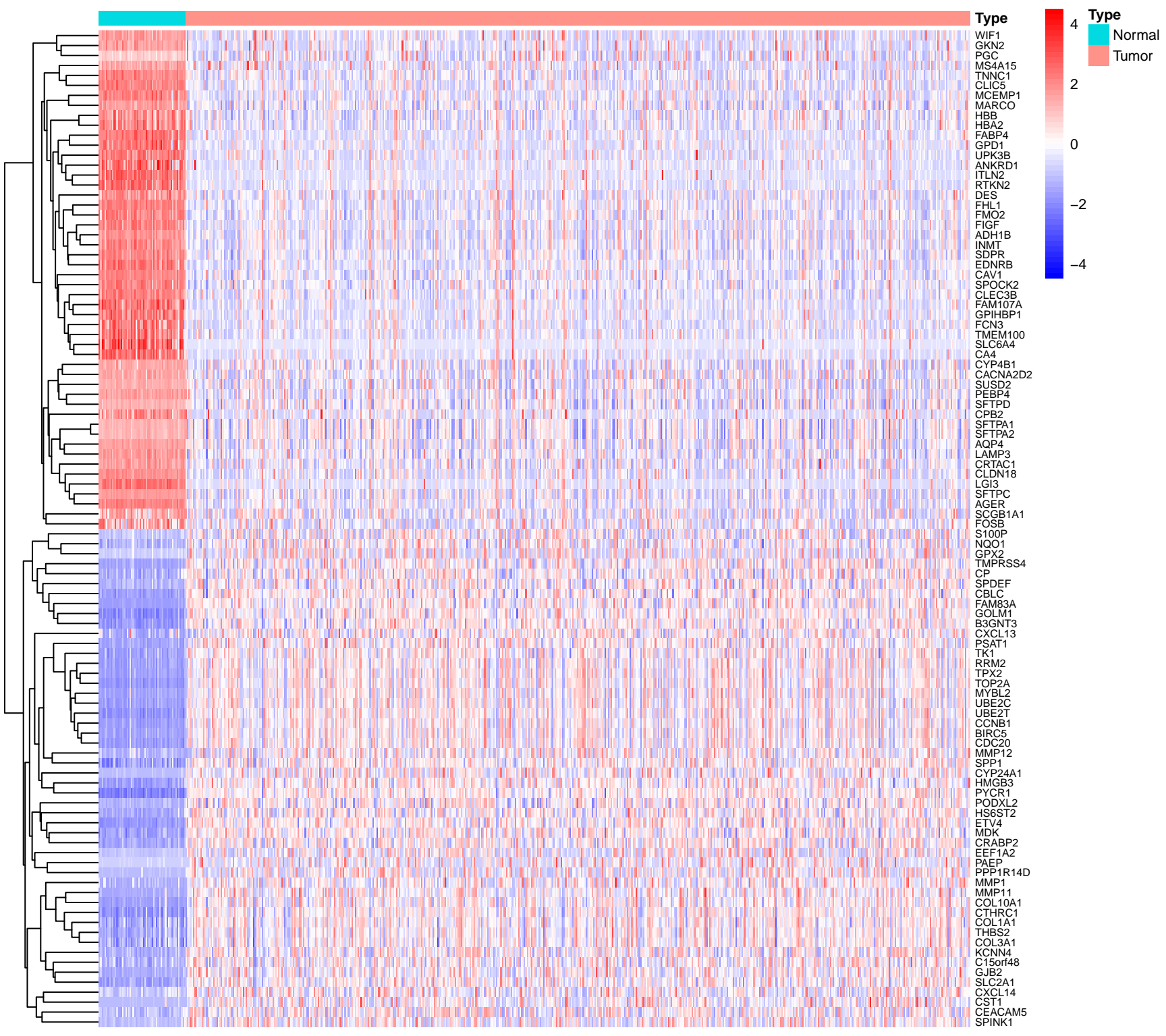

Supplement: Supplementary file 12 [file DataSheet_12.pdf]

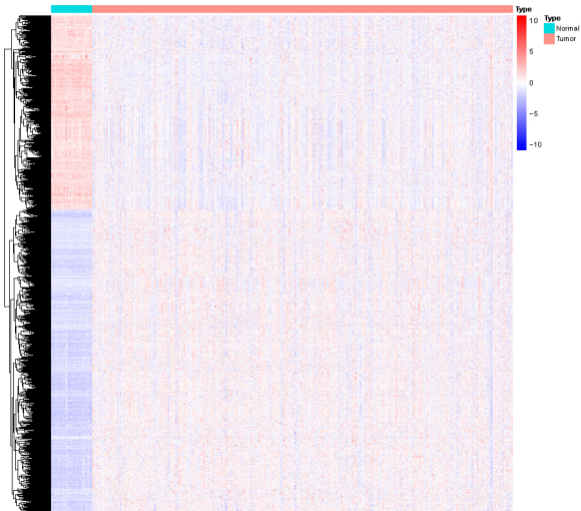

Supplement: Supplementary file 13 [file DataSheet_13.pdf]

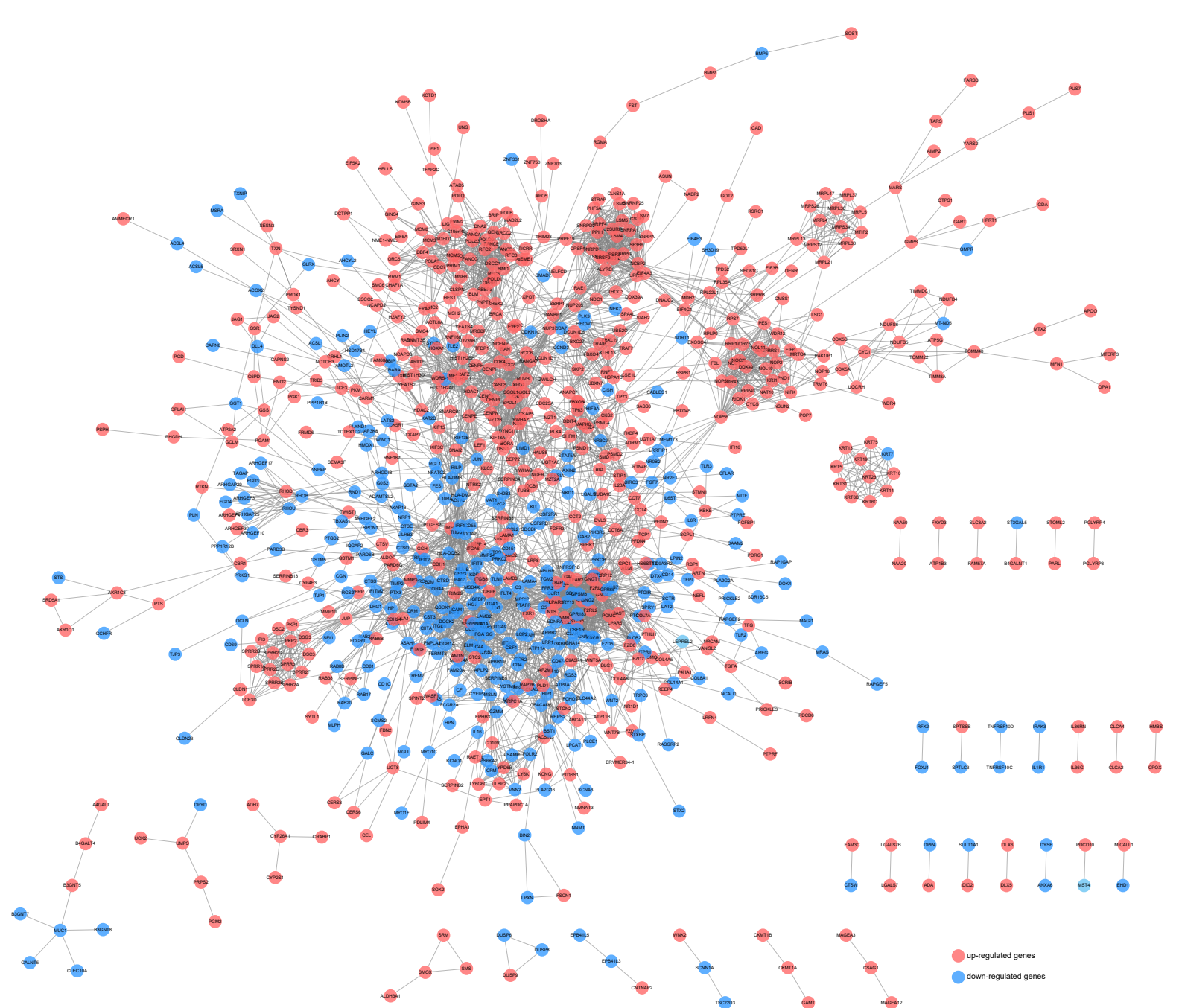

Supplement: Supplementary file 14 [file DataSheet_14.pdf]
